# Serum amyloid A proteins reduce bone mass during mycobacterial infections

**DOI:** 10.3389/fimmu.2023.1168607

**Published:** 2023-04-21

**Authors:** Ana Cordeiro Gomes, Daniela Monteiro Sousa, Tiago Carvalho Oliveira, Óscar Fonseca, Ricardo J. Pinto, Diogo Silvério, Ana Isabel Fernandes, Ana C. Moreira, Tânia Silva, Maria José Teles, Luísa Pereira, Margarida Saraiva, Meriem Lamghari, Maria Salomé Gomes

**Affiliations:** ^1^i3S – Instituto de Investigação e Inovação em Saúde, Universidade do Porto, Porto, Portugal; ^2^IMBC – Instituto de Biologia Molecular e Celular, Instituto de Ciências Biomédicas de Abel Salazar, Universidade do Porto, Porto, Portugal; ^3^INEB – Instituto Nacional de Engenharia Biomédica, Universidade do Porto, Porto, Portugal; ^4^Mestrado em Bioquímica Clínica, Universidade de Aveiro, , Aveiro, Portugal; ^5^Instituto de Ciências Biomédicas de Abel Salazar, Universidade do Porto, Porto, Portugal; ^6^IPATIMUP – Instituto de Patologia e Imunologia Molecular da Universidade do Porto, Porto, Portugal; ^7^CHUSJ – Centro Hospitalar de São João, Porto, Portugal; ^8^EPIUnit, ISPUP - Instituto de Saúde Pública da Universidade do Porto, Porto, Portugal

**Keywords:** inflammation, bone loss, mycobacteria, tuberculosis, SAA

## Abstract

**Introduction:**

Osteopenia has been associated to several inflammatory conditions, including mycobacterial infections. How mycobacteria cause bone loss remains elusive, but direct bone infection may not be required.

**Methods:**

Genetically engineered mice and morphometric, transcriptomic, and functional analyses were used. Additionally, inflammatory mediators and bone turnover markers were measured in the serum of healthy controls, individuals with latent tuberculosis and patients with active tuberculosis.

**Results and discussion:**

We found that infection with *Mycobacterium avium* impacts bone turnover by decreasing bone formation and increasing bone resorption, in an IFNγ- and TNFα-dependent manner. IFNγ produced during infection enhanced macrophage TNFα secretion, which in turn increased the production of serum amyloid A (SAA) 3. *Saa3* expression was upregulated in the bone of both *M. avium*- and *M. tuberculosis*-infected mice and SAA1 and 2 proteins (that share a high homology with murine SAA3 protein) were increased in the serum of patients with active tuberculosis. Furthermore, the increased SAA levels seen in active tuberculosis patients correlated with altered serum bone turnover markers. Additionally, human SAA proteins impaired bone matrix deposition and increased osteoclastogenesis *in vitro*. Overall, we report a novel crosstalk between the cytokine-SAA network operating in macrophages and bone homeostasis. These findings contribute to a better understanding of the mechanisms of bone loss during infection and open the way to pharmacological intervention. Additionally, our data and disclose SAA proteins as potential biomarkers of bone loss during infection by mycobacteria.

## 1 Introduction

Bone loss has been reported during several inflammatory syndromes (1), but the underlying mechanisms remain elusive. Mycobacterial infections are good models to elucidate the molecular cross-talk between the immune response and bone homeostasis. During *in vivo* experimental infection with *Mycobacterium avium*, we noticed that the long bones obtained from infected mice were more brittle and fragile than those obtained from non-infected mice. The presence of mycobacteria in the bone has been traced back to thousands of years ago (2). Mendelian susceptibility to mycobacterial disease is associated with a higher frequency of bone involvement (3). Furthermore, previous mycobacterial infection, even without bone involvement, increases the risk of osteopenia and osteoporosis (4, 5).

A regulated balance between bone degradation and formation maintains an adequate bone mass, conferring the necessary rigidity and strength to the skeleton, to bear weight and avoid low impact fractures. Besides bone forming (osteoblasts and osteocytes) and bone degrading (osteoclasts) cells, bone remodeling is also influenced by immune mediators (1, 6). In this context, we and others have previously shown that mycobacterial infection skews the bone marrow hematopoietic development with important immune and hematological consequences (6–10). Therefore, the infection of bone cells themselves or the immune modulation of the bone marrow niche during infection may disrupt bone mass (6).

In this study, we investigated the mechanisms underlying reduced bone mass during mycobacterial infection to shed light on how the immune system and bone homeostasis articulate. We combined morphometry, transcriptomic and functional *in vitro* and *in vivo* assays to study how infections with the non-tuberculous mycobacteria, *M. avium*, impacted the bone. We established a mechanistic network centered in macrophages and linking IFNγ, TNFα and serum amyloid A (SAA) proteins, which combined to dysregulate bone turnover. The production of SAA by macrophages was confirmed upon direct administration of TNFα to macrophages and during aerosol infection with *M. tuberculosis*. Finally, we showed a systemic increase of SAA levels in patients with active tuberculosis, which positively correlated with altered bone turnover markers measured in the plasma. Our study advances our understanding on how the immune response to infection impacts bone loss and reveals potential drug targets and clinical biomarkers for this. These findings are particularly important in tuberculosis disease, given the enormous number of cases in the world (11), combined with an aging population more susceptible to bone disease.

## 2 Materials and methods

### 2.1 Bacteria

*Mycobacterium avium* 25291 SmT (obtained from the American Type Culture Collection, Manassas, VA) was grown at 37°C in Middlebrook 7H9 broth (BD Difco, USA), supplemented with 0.05% (v/v) of Tween 80 (Sigma, St. Louis, MO) and 10% (v/v) ADC (Albumin-Dextrose-Catalase). *M. avium* were collected during the exponential growth phase, centrifuged, washed twice with saline containing 0.04% (v/v) Tween 80, re-suspended in the same solution, and briefly sonicated at low power to disrupt bacteria clumps. *M. tuberculosis* HN878 (clinical isolate) was grown to midlog phase in Middlebrook 7H9 broth supplemented with 0.05% (v/v) Tween80, and 0.5% (v/v) glycerol. Aliquots were prepared and stored at -80°C, prior to CFU determination or until needed. Just before use, an aliquot was thawed and diluted to the appropriate concentration. Work with *M. avium* and *M. tuberculosis* was performed under ABSL-2 or 3 facilities, respectively.

### 2.2 Recombinant proteins

Recombinant M-CSF, RANKL, TNFa, IFNg were from BioLegend. Recombinant apoSAA was from PrepoTech. All the recombinant proteins had endotoxin levels inferior 0.1 EU/µg protein.

### 2.3 Mice

C57BL/6J, *Ifng*^-/-^, MIIG, and *Tnfa*^-/-^ mice were bred and housed under specific pathogen-free conditions at Instituto de Investigação e Inovação em Saúde (i3S) animal facility. Mice were kept inside individually ventilated cages with HEPA filters and fed with sterilized food and water *ad libitum*. Mice were maintained on a 12:12 light cycle at 45-65% humidity and provided ad libitum water and standardized synthetic diet (Envigo Teklad Global Rodent Diet, 2014S). Animal experiments were approved by the i3S Animal Ethics Committee and licensed by Portuguese Competent Authority (DGAV), in July 6^th^, 2016 with reference 0421/000/000/2016. All animals were handled in strict accordance with good animal practice as defined by national authorities (DGAV, Decreto-Lei 113/2013, August 7^th^) and European Directive 2010/63/EU. The i3S animal facility is AAALAC accredited and follows the Guide for the Care and Use of Laboratory Animals, principle of the Three R’s, to replace, reduce, and refine animal use for scientific purposes, as well as FELASA recommendations.

### 2.4 Human samples

The study protocol leading to serum collection was approved by the Portuguese *Comissão de Ética para a Saúde da ARS Norte* (#T792). To ensure confidentiality, each case was anonymized by the assignment of a random identification number. Participants were enrolled at a TB clinic in Porto and provided informed consent. Experiments were conducted according to the principles expressed in the Declaration of Helsinki. IGRA-positive individuals were assigned to the latent tuberculosis (LTBI) or the active tuberculosis group based on clinical examination and thoracic imaging, according to the guidelines issued by the Portuguese Ministry of Health - Directorate General of Health.

Additionally, 10 serum samples were obtained from blood donors at the Clinic Hematology Service of Centro Hospitalar Universitário do Porto (CHUP) in the same metropolitan area. These subjects are voluntary blood donors that are screened according the Portuguese National Guidelines (Norma n.° 009/2016, updated on March 19^th^ 2021) to exclude active signs of disease and comorbidities. **Table S1** indicates the population demographics.

### 2.5 *In vivo* infection

8- to 10-week-old mice were infected with 1 million CFU of *M. avium* 25291 SmT intravenously (iv) into one of the lateral tail veins. Control groups were injected with the same volume of saline solution by the same route. At 8-weeks p.i., mice were anesthetized using an isoflurane chamber. Blood was collected by retro-orbital puncture under deep anesthesia, immediately before euthanasia. Long bones were also collected. For *M. tuberculosis* experiments, mice were infected *via* the aerosol route using an inhalation exposure system, calibrated to deliver ~200 colony forming units to the lung. The initial bacterial load was determined by quantification of colony forming units in the lungs three days after infection. Infected mice were monitored regularly for signs of illness such as wasting, piloerection and hunching. At 27 days post infection, mice were euthanized by CO_2_ inhalation and long bones were collected. Long bones from aged-matched non-infected mice collected at the same time and used as controls.

### 2.6 Bone morphometric analysis by microCT

Tibias were harvested, cleaned of muscle, fixed in 4% (v/v) paraformaldehyde at 4°C, overnight, and scanned at 70Kv, 200 μA with 0.5 mm aluminum filter, and an isotropic resolution of 6 μm, using SkyScan 1276 System (Brucker, Belgium). The obtained projection images were reconstructed with NRecon software, followed by bone alignment along the sagittal axis using DataViewer software. Morphological analysis of both cortical and trabecular bone were performed with CTAnalyser software, following the recommended guidelines (12). Cortical analysis were carried out in 150 slices at the diaphysis (700 slices away from distal growth plate). 3D images of representative samples were generated using CTVol software.

### 2.7 2D Histomorphometry analysis

Tibias/femurs were cleaned from surrounding soft tissue and fixed in 10% (v/v) PFA for 24 hours and rinsed in phosphate buffered saline (PBS) solution at 4°C. Samples were then dehydrated in serial ethanol solutions (50–100% v/v) for 3 days each, cleared in xylol for 24 hours and further embedded in methyl methacrylate. Undecalcified 7 µm sagittal cuts were stained with modified Masson-Goldner Trichrome, and processed for static histomorphometric analysis as described elsewhere (13), using OsteoMeasure software (OsteoMetrics, Decatur, GA, USA). All histomorpometric analysis were performed by a single blinded operator. Digital images were obtained using NanoZoomer 2.0HT (Hamamatsu) and representative images were acquired.

### 2.8 Dynamic *in vivo* bone labeling

Double labeling with xylenol orange and calcein was used to study bone turnover during *M. avium* infection (14). Thus, 6 weeks post-infection, a saline solution of xylenol orange (Sigma-Aldrich, USA) was subcutaneously administered at a dose of 80 mg/kg. Two weeks later, a saline solution of calcein green (Sigma-Aldrich, USA) at a dose of 15 mg/kg was administered by the same route as xylenol orange. Three days later (at 8 weeks post-infection), mice were euthanized, and femurs and tibias were collected, fixed in 4% (v/v) paraformaldehyde (BioOptica, Italy) at 4°C, overnight. Then, the bones were washed 3 times with PBS1x for 10 minutes, followed by dehydration with PBS1x/30% (w/v) sucrose, at 4°C, overnight. Long bones were embedded in OCT (ThermoFisher, UK), snap-frozen in an ethanol/dry ice bath, and 20-µm sections were cut using the Kawamoto method (15), using a Leica CM 3050S cryostat (Leica Biosystems, Portugal). Sections were kept at -20°C, inside a silica gel chamber, for at least 12 hours. Then, slices were warmed to room temperature inside a silica gel chamber and mounted in a 30% (v/v) glycerin solution. Images were acquired on a Leica SP8 confocal microscope (Leica Microsystems, Germany), using LASX software (Leica Microsystems, Germany). Images were analyzed using the measurement tool of Imaris Software (Oxford Instruments, UK).

### 2.9 Immunofluorescence of femur whole mounts

Long bones were collected from infected mice at 8 weeks post-infection and from littermate non-infected controls, fixed, dehydrated, and embedded in OCT, as described before. Then, bones were trimmed in a cryostat to expose the medullary cavity, and OCT was removed by melting at room temperature. After 3 washes with PBS for 10 minutes, to block the non-specific binding and to permeabilize, whole mounts were incubated for 1 hour at room temperature with PBS containing 20% (v/v) fetal bovine serum and 0,5% (v/v) Triton X-100 (Sigma Aldrich, USA). Afterwards, whole mounts were stained for 3 days, at room temperature, with rabbit anti-TRAP antibody (1:50 dilution (v/v), Abcam, UK). Whole mounts were washed in PBS for 10 minutes, three-times, and stained with Alexa 647 anti-rabbit antibody (1: 500 dilution (v/v), Jackson ImmunoResearch, UK) and DAPI (Sigma-Aldrich) for 3 days, at room temperature and protected from the light. After 3 washes in PBS for 10 minutes, whole mounts were transferred to an imaging chamber (ibidi GmbH, Germany) containing 30% (v/v) glycerin solution and imaged on a Leica SP8 confocal microscope (Leica Microsystems, Germany). Bone surface expressing TRAP was quantified using Imaris Software (Oxford Instruments, UK).

### 2.10 Histology

Femurs were fixed in 10% (v/v) neutral buffered formalin solution overnight at 4°C, in EDTA/Glycerol (v/v) solution for 3 weeks at 4°C, and then included in paraffin blocks. 5-μm thick sections were cut, deparaffinized and processed through downgraded alcohols, and rehydrated.

#### 2.10.1 Immunohistochemistry of F4/80 and Ziehl-Neelsen stain

Sections were deparaffinized and processed as described before. To unmask the antigenic epitope, sections were incubated in citrate buffer pH 6.0 (Thermo Fisher Scientific) in a steamer for 20 minutes, allowed to cool for 10 minutes at RT, followed by enzymatic digestion with 0.05% (v/v) Trypsin (Gibco) in a humidified chamber at 37°C for 30 minutes. Enzymatic reaction was stopped with incubations in cold water for 5 minutes, twice. After blocking of endogenous peroxidase activity using Ultravision hydrogen peroxide block (Thermo Fisher Scientific), endogenous biotin was blocked (Lab Vision Avidin biotin Blocking Solution, Thermo Fisher Scientific). Unspecific binding sites were blocked with Normal Goat Serum (1:5 dilution (v/v), DAKO) in antibody diluent (Thermo Fisher Scientific) at RT for 30 minutes. Rat anti-mouse F4/80 antibody (clone BM8, Biolegend) was added to the sections and incubated in a humidified chamber overnight at 4°C. Then, sections were stained with a biotinylated goat anti-rat IgG (Enzo) in a humidified chamber at room temperature for 30 minutes, followed by incubation with Streptavidin-HRP (Vector Laboratories) in a humidified chamber at RT for 30 minutes. DAB Quanto (Thermo Fisher Scientific) was added to allow the color development for 3 minutes. Slides were stained with Carbol Fuchsin and then heated and rinsed off in tap water. Then, 1% (v/v) of hydrochloric acid in methanol was added to slides. Slides were counter stained with Hematoxylin, rinsed twice with water, dehydrated (reverse of downgraded alcohols and deparaffinization), and coverslipped in Entellan. Sections were scanned in D-sight Plus f2.0 and analyzed with D-Sight viewer.

### 2.11 Flow cytometry

Cells were isolated from bone marrow of long bones by flushing in DMEM supplemented with 2% (v/v) heat-inactivated bovine fetal serum, 5% (v/v) HEPES, and PenStrep (all from Invitrogen). Cells were counted using the trypan blue (Sigma-Aldrich) exclusion assay.

#### 2.11.1 Myeloid cell analysis

One million BM cells were stained with anti-Gr-1 (clone RB6-8C5, BioLegend), anti-CD115 (clone AFS98, BioLegend), anti-F4/80 (clone BM8, BioLegend) and anti-CD169 (clone 3D6.112, BioLegend). for 30 minutes, on ice. Cells were washed twice at 2000 rpm for 2 minutes and fixed with 2% (v/v) PFA for 10 minutes at room temperature. Cells were gated as previously described (16). Neutrophils: Gr-1^hi^ CD115^-^; Monocytes: Gr1^lo^ CD115^+^ F4/80^+^; Macrophages: Gr1^lo^ CD115^-^ F4/80^+^ CD169^+^.

#### 2.11.2 Stromal cells analysis

The isolation of bone marrow stromal cells was performed as described before (17). Briefly, long bones were carefully flushed using HBSS supplemented with 2% (v/v) heat-inactivated fetal bovine serum, PenStrep, HEPES and 200 U/mL Collagenase IV (Sigma-Aldrich). The flushed bone marrow was then digested at 37°C for 20 minutes. Cells clumps were dissociated by gentle pipetting, followed by a further incubation for 10 minutes at 37°C. Cells were then washed with HBSS/2% (v/v) FBS, spun at 1000 rpm for 7 minutes and stained. LEPR staining was performed using biotin-conjugated anti-LEPR (R&D systems) for 1 hour, on ice. The remaining staining using PE-conjugated anti-CD45 (clone 30-F11, BioLegend) and anti-TER119 (BioLegend) and APC-conjugated anti-CD31 (clone 391, BioLegend) and anti-CD144 (BV13, BioLegend) were done for 20 min, on ice, followed by 2 washes at 1000 rpm for 7 minutes. Finally, cells were washed and fixed with 2% (v/v) PFA for 10 minutes at room temperature. Cells were analyzed using a BD FACS Canto II flow cytometer and FlowJo.

### 2.12 RNA isolation and quantitative real time PCR

For *M. avium*-infected mice and littermate non-infected mice, bones were harvested and snap frozen in liquid nitrogen. Bones were crushed in a liquid nitrogen bath and total RNA was isolated using RNA Mini Kit (Invitrogen), according to manufacturer instructions. For *M. tuberculosis*-infected mice bone marrow was flushed from femurs and tibias and extracted with Trizol. cDNA was then synthetized using NZY First-Strand cDNA synthesis kit (NZYTech). Primers sequences are in **Table S2**.

### 2.13 Targeted transcriptomic analysis

Whole bone total RNA was isolated from C57BL/6J mice at 8 weeks post-infection and from littermate non-infected mice, as described above. The transcriptomic laboratorial processing was performed by the Genomics Core Facility at i3S (Porto, Portugal). Briefly, RNA concentration was measured using Qubit3.0 fluorometer and total RNA integrity number was determined using the Agilent 2100 Bioanalyzer. Libraries were constructed according to Ion AmpliSeq™ Transcriptome Mouse Gene Expression Kit protocol (Thermo Fisher Scientific, USA) and pooled with each sample ligated to a unique barcode. The pooled libraries were processed on Ion Chef™ System and the resulting 540™ chip was sequenced on Ion S5™ XL System. FASTQ and/or BAM files were generated using the Torrent Suit plugin FileExporter v5.12.

The statistical analysis and graphical representations were performed in R studio version 1.4.1106. The quality control of the expression profiles of triplicates of each experimental group were investigated through Principal Component Analysis (PCA). Differentially expressed genes in the bone between infected and non-infected bones were identified by the R package DESeq2 package v1.24 with default parameters (18), considering an adjusted p-value threshold below 0.05 and a log_2_FoldChange>0.5. Clustering and heatmap representation of these significantly expressed genes, between uninfected and infected tests, were obtained using the R package heatmap version 1.0.12 package. Pre-ranked pairwise gene-set enrichment analyses (GSEA) were conducted in GSEA software for the Gene Ontology (GO) (biological process or BP, cellular component or CC, and molecular function or MF) and KEGG pathway datasets, after converting the mouse genes in the homologous human genes by using information from the Mouse Genome Informatics Web Site (19). The resulting enriched pathways were ordered by the normalized enrichment score (NES) and the false discovery rate (FDR) q-value. Top significantly enriched pathways (FDR < 0.05) were further explored based on information contained in the publicly available database of Ingenuity (https://targetexplorer.ingenuity.com/index.htm; Qiagen, Hilden, Germany). Cytoscape v3.9.1 (20) with the Enrichment Map plugin (21) was used for the interpretation and visualization of the GSEA results. The enrichment map was generated within enriched gene gets that passed the significance threshold of FDR Q<0.05.

### 2.14 Measurement of SAA, TNFα and CTX-I by ELISA

Blood was collected from the orbital vein to non-heparinized tubes and let to clot and spun at high speed to collect serum. Serum levels of SAA3, TNFα and CTX-I were measured using the mouse SAA-3 ELISA kit (Sigma-Aldrich), LEGEND MAX™ Mouse TNF-α ELISA Kit (BioLegend) and the RatLapsTM EIA CTX-I (Immunodiagnostic Systems, Denmark), following the manufacturer’s instruction.

Human peripheral blood was collected by venous puncture and plasma was recovered. SAA and CTX-I were measured using the human SAA 1 and 2 ELISA (Sigma-Aldrich) and Serum Crosslaps (CTX-I) ELISA (ImmunoDiagnosticSystems, Denmark), following the manufacturer’s instruction. Circulating levels of cytokines/chemokines and bone biomarkers were determined using the Human Cytokine/Chemokine Panel A 48-Plex Discovery Assay^®^ Array (HD48A) and Human Bone 13-Plex Discovery Assay^®^ Array (HDBN13), respectively (Eve Technologies, Calgary, Canada).

### 2.15 Differentiation of bone marrow-derived macrophages

Bone marrow was harvested from the long bones of C57BL/6 mice by flushing with Hank’s Balanced Salt Solution (HBSS, Gibco, UK). Cells were cultured overnight in DMEM containing 10% (v/v) fetal bovine serum, 1% (v/v) pyruvate, 1% (v/v) glutamine, 1% (v/v) HEPES, and 10% (v/v) L929 cell-conditioned medium (LCCM), at 37°C/5% CO_2_. Non-adherent cells were collected and then plated at a cellular density of 5x10^5^ cells/mL. Cells were cultured in DMEM containing 10% (v/v) fetal bovine serum, 1% (v/v) pyruvate, 1% (v/v) glutamine, 1% (v/v) HEPES, and 10% (v/v) L929 cell-conditioned medium (LCCM), at 37°C/5% CO_2_. At day 4 of culture, 10% (v/v) LCCM was added and at day 7, the medium was renewed. At the 10^th^ day, cell culture media was collected to obtain non-infected bone marrow-derived macrophage conditioned media (NI BMDM CM). Then, cells were *in vitro* infected with 5x10^5^ CFU/mL of *M. avium* 25291 SmT, for 4 hours at 37°C/5% CO_2_. Afterwards, cells were washed 4 times with warm HBSS and then incubated at 37°C/5% CO_2_ for 5 days. For TNFα and IFNγ treatments, a concentration of 10 ng/mL and 16ng/mL, respectively, was added to the cultures for 3 days post-infection. At day 5 post-infection, cell culture medium was removed and filtered to obtain *M. avium*-infected bone marrow-derived macrophages conditioned medium (*Mav* BMDM CM). Conditioned media were stored at -80°C until used. RNA was harvested from infected and non-infected cultures using RNA Mini Kit (Invitrogen). Gene expression was determined as previously described.

### 2.16 Osteoclast differentiation assays

Hematopoietic progenitors were harvested from the bone marrow by flushing the long bones with αMEM supplemented with 10% (v/v) fetal bovine serum. After red blood cell lysis, cells were cultured overnight in αMEM supplemented with 10% (v/v) fetal bovine serum and 10 ng/mL M-CSF, at 37°C/5% CO_2_. Non-adherent cells were collected and then cultured at a cell density of 7.5x10^5^ cells/mL, with 100ng/mL RANKL (BioLegend), 100 ng/mL M-CSF (BioLegend), and 50% (v/v) NI or *Mav* BMDM CM. Media was renewed every other day. At the 7^th^ day of culture, cells were fixed with 4% (v/v) paraformaldehyde (BioOptica, Italy) for 10 minutes, at room temperature, and permeabilized with 0.1% (v/v) Triton X-100 in PBS 1x for 15 minutes at room temperature. Nuclei were labelled using DAPI, and cytoplasm was labeled with HCS CellMask (Invitrogen, USA). Imaging was performed using IN CELL Analyzer 2000 microscope (GE Healthcare, USA). Osteoclasts were manually quantified using Image J and Microsoft Excel (Microsoft Corporation, USA). The number of nuclei per cell is usually considered to reflect the degree of osteoclast differentiation (22). In this study, an osteoclast was defined as a multinucleated cell with more than 3 nuclei per cell. Four ranges were defined: 3-4 nuclei per cell, 5-10 nuclei per cell, 11-20 nuclei per cell and more than 20 nuclei per cell. The frequency of each class was calculated. To determine osteoclast resorptive capacity, osteoclast precursors were seeded on top of commercially available bovine cortical bone slices (boneslices.com, Denmark). At day 7, cells were lysed by incubation with water. Bone slices were washed and stained with Toluidine Blue. The total number of pits and trenches – the two types of resorptive activity attributed to osteoclasts- per slice was enumerated using an optical microscope.

### 2.17 Osteoblast cultures

Bone marrow stromal cells were flushed from the long bones C57BL6 mice with αMEM supplemented with 10% (v/v) fetal bovine serum, plated in 75 cm^2^ flasks and cultured for 7 days. Medium was renewed every 2-3 days to remove hematopoietic cells in suspension. Primary osteoprogenitor cells were harvested at pre-confluence using trypsin solution and cell viability was determined by the Trypan Blue Exclusion assay. Then, cells were plated at a 5x10^5^ cells/mL, under osteogenic conditions (MesenCult Osteogenic Stimulatory Kit, Stem Cell Technologies, USA). Medium was renewed every 3 days. At day 12 of culture under osteogenic conditions, cells were fixed with 4% (v/v) paraformaldehyde for 10 minutes, at room temperature, and stained with alizarin-red staining solution (Sigma-Aldrich, USA) for 30 minutes, at room temperature with gentle shaking. Then, calcium deposits were eluted with 10% (w/v) cetypyridinium chloride in sodium phosphate (pH 7.0) and quantified at 570 nm.

### 2.18 Statistics

Data was tested for normality using the Kolmogorov-Smirnov test and Shapiro-Wilk test. When data followed a normal distribution, t test or ANOVA were used to compare the experimental groups. For non-parametric data, Mann-Whitney test was used. Statistical analysis was performed using GraphPad Prism 9 (GraphPad Software, CA, USA). A p value <0.05 was considered significant.

## 3 Results

### 3.1 Mycobacterial infection reduces bone mass and mineral density due to both increased degradation and decreased formation

**Figure 1:**
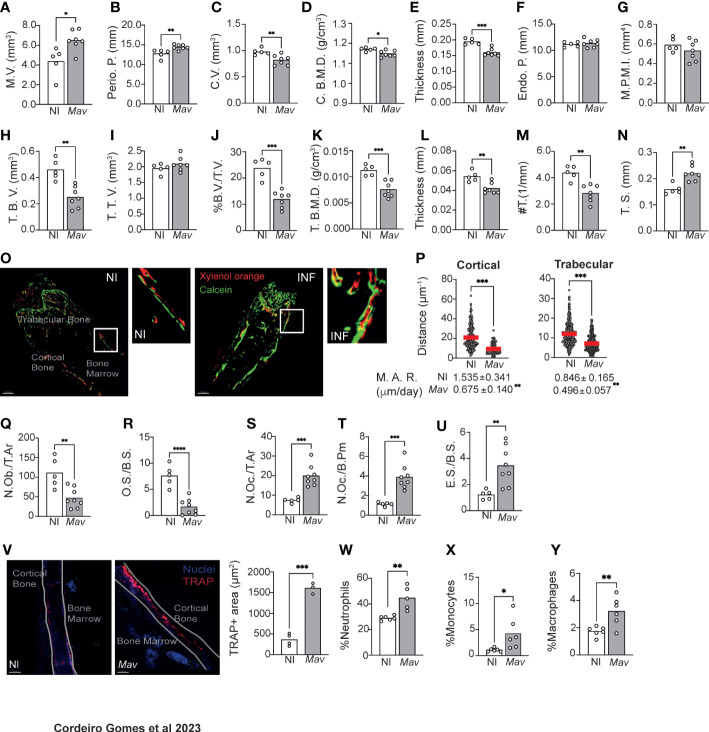
*Mycobacterium avium* infection alters bone turnover, reducing bone mass. **(A–G)** Measurement of the marrow volume (M.V., **(A)**, periosteal perimeter (Perio. P., **(B)**, cortical volume (C.V., **(C)**, cortical bone mineral density (C. B. M. D., **(D)**, cortical thickness **(E)**, endocortical perimeter (Endo. P., **(C)**, and polar mean moment of inertia (M. P. M. I., **(G)** in *M. avium*-infected mice (*Mav* bars) compared to non-infected littermate controls (NI bars). **(H–N)** Measurement of the trabecular bone volume (T.B.V., **(H)**, trabecular tissue volume (T.T.V., **(I)**, ratio of bone volume to tissue volume (%B.V./T.V., **(J)**, trabecular bone mineral density (T. **(B)** M. D., **(F)**, trabecular thickness **(L)**, trabecular number (#T, **(M)** and trabecular separation (T.S., **(N)** in *M. avium*-infected mice (*Mav* bars) and non-infected controls (NI bars).N=5-7 mice per experimental group; data representative of two independent experiments. Bars indicate average of the group, and each circle depicts each individual mouse analyzed. *, p<0.0,5, ** p<0.01, ***, p<0.001 by Mann-Whitney test. **(O)** 20-μm-thick sections of femoral bone labeled with xylenol orange (red) and calcein (green). Scale bar: 100μm. **(P)** Distance between xylenol orange and calcein layers on the bone surface of non-infected (NI) and infected (*Mav*) mice. Bellow the graphs, the mineral apposition rate (M.A.R) is indicated. N=5-6 mice per experimental group. Red lines indicate average, and circles depict each measurement. ***, p<0.001 by unpaired Student’s *t* test. **(Q–U)** Histomorphometry analysis of the long bones of *M. avium*-infected mice (*Mav* bars) compared with non-infected controls (NI bars). Osteoblast number per total area (N.Ob/T.Ar, **(Q)**, Osteoid surface per bone surface (OS/BS, **(R)**, Number of osteoclast per total area (N.Oc/T.Ar, **(S)**, Number of osteoclasts per bone perimeter (N.Oc./B.Pm., **(T)**, Eroded surface per bone surface (ES/BS, **(U)**. N=5-8 mice per experimental group. Representative of two independent experiments. Bars indicate the average; circles depict each individual mouse. ** p<0.01, ***, p<0.001 by Mann-Whitney test. **(V)** Quantification of the bone area expressing TRAP. TRAP-expressing bone surface was identified in the bones of infected (*Mav*) and non-infected (NI) mice and the area of those regions was then measured. TRAP staining in red; nuclei in blue; white line shows the limits of the bone; NI, non-infected; *Mav*, infected for 8 weeks; Scale bar: 150μm. Bars represent the average of the experimental group; each dot corresponds to one individual animal. **(W-Y)** Frequency of neutrophils (Gr-1^hi^ CD115^-^; **(W)**, monocytes (Gr1^lo^ CD115^+^ F4/80^+^; **(X)**, and macrophages (Gr1^lo^ CD115^-^ F4/80^+^ CD169^+^; **(Y)** in the bone marrow. N=6 mice per experimental group. Representative of three independent experiments. Bars indicate the average; circles depict each individual mouse. *, p<0.0,5, ** p<0.01, *** p<0.001, **** p<0.0001 by unpaired Student’s *t* test.

Our first aim was to comprehensively characterize the alterations induced in the bone during *M. avium* infections. For that, C57BL/6J mice were infected with *M. avium* through the iv route (23). Eight weeks later several parameters in the cortical and trabecular bone were quantified by micro-computerized tomography (microCT, **Figure S1A–C**). Even though the marrow volume (M.V.) and periosteal perimeter (Perio. P.) were increased in the cortical bone (**Figures 1A, B**), the cortical volume (C.V.), mineral density (C. B.M.D.) and thickness were reduced by infection (**Figures 1C–E**). Endocortical perimeter (Endo. P.) and mean polar moment of inertia (MPPI) were not altered (**Figures 1F, G**). Additionally, the trabecular bone volume (T.B.V.) was decreased (**Figure 1H**) whereas the trabecular tissue volume (T.T.V.) was not altered (**Figure 1I**), which resulted in a reduction of the ratio trabecular bone volume to tissue volume (%B.V./T.V., **Figure 1J**). Also reduced in infected mice were the trabecular mineral density (T. B.M.D.), and the trabecular thickness and number (#T., **Figures 1K–M**), with the concomitant increase in the trabecular separation (T. S.; **Figure 1N**).

Given these alterations suggestive of bone loss during infection, a dynamic histomorphology analysis was performed (14). Bone surface was fluorescently labeled *in vivo* by the injection of xylenol orange and calcein green at 6- and 8-weeks post-infection, respectively. Mice were sacrificed 3 days after the latter, and 20-μm bone sections were analyzed by confocal microscopy. In non-infected mice, the two fluorochrome labels were detected in the bone and were visibly separated by non-labeled (newly formed) bone tissue. However, in the infected bones, even though both calcein and xylenol orange were detected, some regions were not labeled with xylenol orange, or the two layers of dyes overlapped (**Figure 1O**). The distance between the xylenol and calcein layers in cortical and trabecular bone, indicative of the degree of bone formation, was measured and found to be significantly decreased in infected as compared to non-infected mice. Concomitantly, the mineral appositional rate (M.A.R.) was significantly decreased in infected mice (**Figure 1P**).

In what regards the cellularity of the bone, we found that infection reduced the number of osteoblasts (N.Ob/T.Ar.) and osteoid volume per bone surface (O.S./B.S.) (**Figures 1Q, R**), as well as the number of osteoblasts lining the bone surface (N.Ob./B.Pm.; **Figure S1D**). The alterations in osteoblast lineage could also be detected at the LEPR^+^ mesenchymal stem cells (MSC) level, whose frequency was increased in the bone marrow of infected mice (**Figure S1E**). LEPR^+^ MSC were described as the major source of osteoblasts and adipocytes in the adult bone marrow (24). The increase in in the frequency of LEPR+ MSC may indicate a blockade in their differentiation towards osteoblasts.

Finally, histomorphology analysis of the bone revealed an increased number of osteoclasts per total area (N.Oc./T.Ar.) and per total bone perimeter (N.Oc./B.Pm., **Figures 1S,T**), which led to an increased eroded bone surface per bone surface (E.S./B.S.; **Figure 1U**). Together these findings indicate that bone loss is happening due to exacerbated osteoclast activity. To test this hypothesis, we measured the area of TRAP+ bone, which is indicative of osteoclast activity as TRAP is an enzyme secreted by osteoclasts during bone resorption. The area of TRAP+ bone surface was increased in infected mice (**Figure 1V**). Furthermore, significantly higher levels of C-terminal telopeptides of Type I collagen (CTX-I) were detected in the serum of infected mice (**Figure S1F**), an indicator of increased bone resorption.

Osteoclasts have a myeloid origin. For this purpose, myeloid populations were identified as previously described (16). We found an increased frequency of neutrophils, monocytes and macrophages are present in the bone marrow of infected mice (**Figures 1W, Y**). These data are in agreement with previous reports that mycobacterial infections skew the differentiation of hematopoietic stem cells towards the myeloid lineage (8–10).

Disseminated *M. avium* infection leads to the colonization of macrophages within the bone marrow parenchyma (23) instead of a direct colonization of bone cells (**Figure S2**), suggesting that the altered bone turnover and reduced bone mass that we now show during infection might be a consequence of the ongoing immune response instead of a direct colonization of bone cells.

### 3.2 IFNγ and TNFα production during *M. avium* infection lead to bone resorption

**Figure 2:**
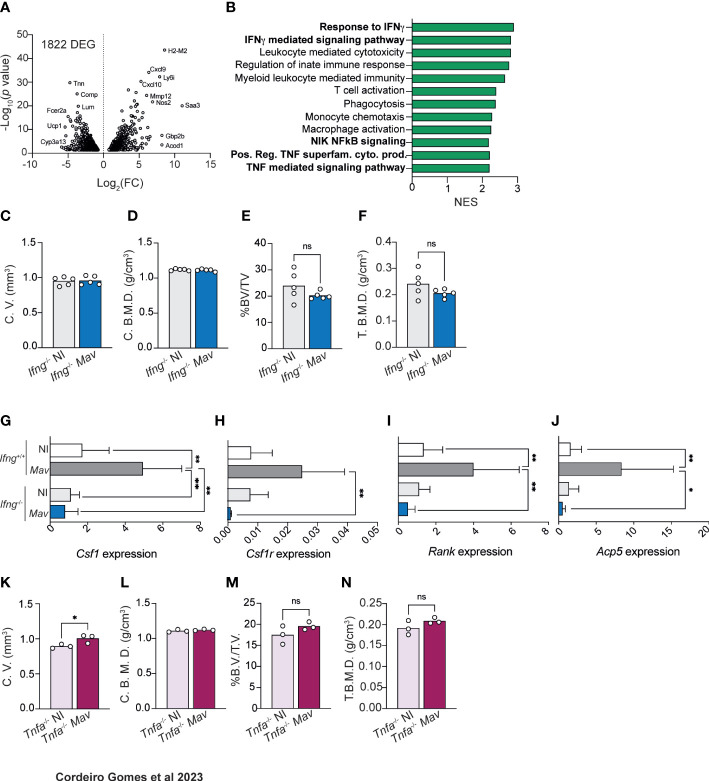
Bone mass reduction during chronic *M. avium* infection is dependent on IFNγ and TNFα production. **(A)** Volcano plot of the differentially (and statistically significant) expressed genes in the bone of *M. avium*-infected mice compared to non-infected littermate control mice. **(B)** Differentially upregulated pathways (from the GO Biological Process dataset) in the bone of infected mice compared with non-infected controls. **(C, D)** Measurement of the cortical volumes (C. V., C), and cortical bone mineral density (C. B. M. D., D) in *M. avium*-infected (*Mav* bars) compared to non-infected mice (NI bars). **(E, F)** Measurement of the ratio of bone volume to tissue volume (%BV/TV, E), and trabecular mineral bone density (T. B. M. D., F). N=5 mice per experimental group; data representative of two independent experiments. Bars indicate average; circles depict each individual mouse analyzed. n.s., p>0.0,5 by Mann-Whitney test. **(G–J)** Expression of *Csf1* **(G)**, *Csf1r* **(H)**, *Rank* **(I)**, *Acp5* **(J)**, in the bones of infected (*Mav* bars) and non-infected (NI bars) *Ifng*^-/-^ and *Ifng*^+/+^ mice. Bars represent the average ± standard deviation of the experimental group. *, p<0.05, ** p<0.01 by two-way ANOVA. **(K, L)** Measurement of the cortical volumes (C. V., K), and cortical bone mineral density (C. B. M. D., L). **(M, N)** Measurement of the ratio of bone volume to tissue volume (%BV/TV, M), and trabecular mineral bone density (T. B. M. D., N). N=3 mice per experimental group; data representative of two independent experiments. Bars indicate average; circles depict each individual mouse analyzed. n.s., *p*>0.05 by Mann-Whitney test.

To gain insight into the molecular mechanisms behind reduced bone mass during mycobacterial infection, we performed targeted RNAseq of whole bone and compared the gene expression between non-infected and infected mice. We found that 1822 genes were statistical and differentially expressed (adjusted *p*-values of ≤ 0.05) in the bone of infected mice (**Figure 2A**). Within the top upregulated differentially expressed genes (DEG) were several immune mediators, such as *Cxcl9*, *Cxcl10* and *Nos2* (**Figure 2A**). Furthermore, pathway-enrichment analysis revealed that the most upregulated (and statistically significant) pathways during *M. avium* infection were “response to IFNγ“ and “IFNγ mediated signaling pathway” (**Figure 2B**, **Figure S3**). Next, we investigated whether this cytokine was also involved in bone loss during infection. For that, *Ifng*^-/-^ mice were infected with *M. avium*. We showed previously that the proliferation of the *M. avium* strain used in this study is not significantly different in *Ifng*^-/-^ as compared to *Ifng*^+/+^ mice (23). MicroCT analysis revealed that most of the alterations in cortical and trabecular bone found in infected of *Ifng*^+/+^ (**Figure 1**) were absent in mice lacking *Ifng* (**Figures 2C–F**; **Figures S1G–P**). Thus, IFNγ is a central piece mediating bone loss during *M. avium* infection.

The analysis also highlighted several pathways related to myeloid and monocytic function, which we hypothesized could be related to osteoclast differentiation (1, 6, 25–27). In fact, *M. avium* infection induced the up-regulation of *Csf1*, *Csf1r*, *Rank*, and *Acp5* in the bones of *Ifng*^+/+^ but not of *Ifng*^-/-^ mice (**Figures 2G–J**). Overall, our data indicate that osteopenia during *M. avium* infection depends on IFNγ production, which mediates the upregulation of genes related to osteoclast formation and activity. These observations agree with previous studies reporting that IFNγ is an indirect promoter of osteoclastogenesis and bone resorption (28).

Besides IFNγ-related pathways, our RNA-Seq data highlighted an upregulation of pathways associated with TNF, NIK and NFkB signaling in the bone of infected mice (**Figure 2B**). Thus, we decided to investigate the contribution of TNFα to the bone alterations induced by *M. avium* infection. In mice lacking the *Tnfa* gene, *M. avium* infection did not generally result in reduction of cortical and trabecular bone mass and mineral density (**Figure 2K–N**; **Figures S1Q–Z**). Of note, cortical bone volume, trabecular bone volume and trabecular total volume were significantly increased in *Tnfa*^-/-^ infected mice (**Figure 2K**; **Figure S2X, Y**). Together, the data suggest that osteopenia during *M. avium* infection requires both IFNγ and TNFα.

### 3.3 Soluble factors produced by *M. avium*-infected macrophages increase osteoclastogenesis

*In vivo* data indicated that IFNγ and TNFα were involved in osteoclastogenesis and bone resorption during infection. We thus decided to use *in vitro* systems to investigate whether these cytokines had a direct effect on osteoclastogenesis.

**Figure 3:**
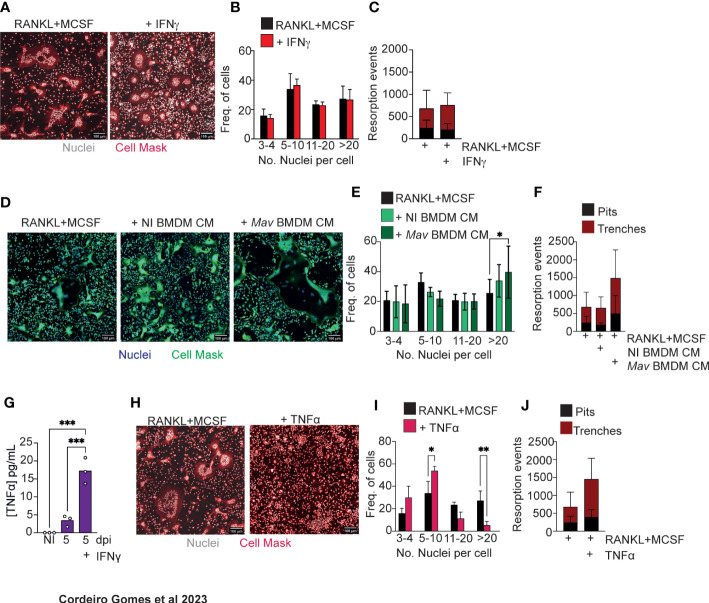
Soluble factors produced by *M. avium*-infected macrophages increase osteoclastogenesis. **(A)** Osteoclast differentiation in the presence of saturating amounts of M-CSF and RANKL (left panel) and with the addition of 1 ng/mL of IFNγ. **(B)** Frequency of multinucleated cells grouped by number of nuclei per cell. Data are pooled from three independent experiments. Bars represent the average ± standard deviation of the three independent experiments. **(C)** Enumeration of pits (black bars) and trenches (red bars). Data are representative of two independent experiments. Bars represent the average ± standard deviation of 4 replicates in each experimental group. **(D)** Osteoclast differentiation in the presence of saturating amounts of M-CSF and RANKL (left panel) and with the addition of conditioned media (CM) from non-infected (NI BMDM, middle panel) and *M. avium*-infected bone marrow-derived macrophages (*Mav* BMDM) (right panel). **(E)** Frequency of multinucleated cells grouped by number of nuclei per cell. Data are pooled from six independent experiments. Bars represent the average ± standard deviation of the six independent experiments. *, p<0.05, by Two-way ANOVA. **(F)** Enumeration of pits (black bars) and trenches (red bars). Data are representative of two independent experiments. Bars represent the average ± standard deviation of 4 replicates in each experimental group. **(G)** Measurement of TNFα in conditioned media from bone marrow derived and *M. avium* infected-macrophages. **(H)** Osteoclast differentiation in the presence of saturating amounts of M-CSF and RANKL (left panel) and with the addition of TNFα (right panel). **(I)** Frequency of multinucleated cells grouped by number of nuclei per cell. Data are pooled from four independent experiments. **(J)** Enumeration of pits (black bars) and trenches (red bars). Data are representative of two independent experiments. Bars represent the average ± standard deviation of the experimental group. *, p<0.05, ** p<0.01, ***p<0.001 by Two-way ANOVA.

Firstly, osteoclasts were differentiated *in vitro* with RANKL and MCSF, the two osteoclastogenesis instructing cytokines. The baseline of differentiation was determined for the condition RANKL and MCSF alone. IFNγ addition to these *in vitro* osteoclast cultures did not alter osteoclastogenesis relatively to baseline conditions (**Figures 3A, B**). Secondly, 3D cultures of osteoclasts on bone slices were performed to determine the bone resorption capacity of the formed osteoclasts. Osteoclasts formed in the presence of IFNγ similarly resorbed bone compared to the cultures without IFNγ addition (**Figure 3C**). These data suggest that the role of IFNγ in bone loss *in vivo* is most likely indirect.

Because mycobacteria reside mostly inside macrophages, we hypothesized that osteoclastogenesis could be modulated by soluble factors produced by infected macrophages in the bone marrow parenchyma. So, we next repeated our *in vitro* experiments supplementing the osteoclast cultures with conditioned media obtained from non-infected or infected BMDM. In the presence of conditioned media from infected macrophages, the frequency of osteoclasts with more than 20 nuclei increased (**Figures 3D, E**). Moreover, in 3D cultures of osteoclasts on top of bone slices, more pits and trenches were formed in the presence of conditioned media from *M. avium*-infected macrophages (**Figure 3F**). Together, our data indicate that the local production of cytokines and other soluble factors by macrophages during infection leads to an increased osteoclast production.

Taking our above findings showing that both IFNγ and TNFα are required as *in vivo* mediators of bone loss, that IFNγ likely acts through indirect mechanisms and that soluble factors produced by infected macrophages induce osteoclast production, we hypothesize that IFNγ may act on macrophages during infection to potentiate TNFα production, which in turn directly or indirectly promotes osteoclast activity. Thus, we firstly measured TNFα production by *M. avium* infected macrophages in the absence or presence of IFNγ. As expected (29), the production of TNFα was elevated in the presence of IFNγ (**Figure 3G**). TNFα treatment of osteoclast cultures reduced the number of nuclei per osteoclast (**Figures 3H, I**), whilst inducing osteoclasts to perform more resorption events, namely trenches (**Figure 3G**), as compared to baseline conditions. Overall, our results indicate that TNFα directly promotes osteoclast activity by increasing the proportion of trenches formed on the bone surface. Contrary to the standard mode of bone resorption (the pit mode), the trenches result from a faster and longer resorption of bone as the osteoclasts move, creating deeper and parallel erosions on the bone surface (30, 31), suggesting a more aggressive resorption mode. Additionally, our data also indicate that other soluble factors in produced by infected macrophages, in addition to TNFα, may impact osteoclast formation.

### 3.4 SAA3 levels increase both locally and systemically during mycobacterial infection

**Figure 4:**
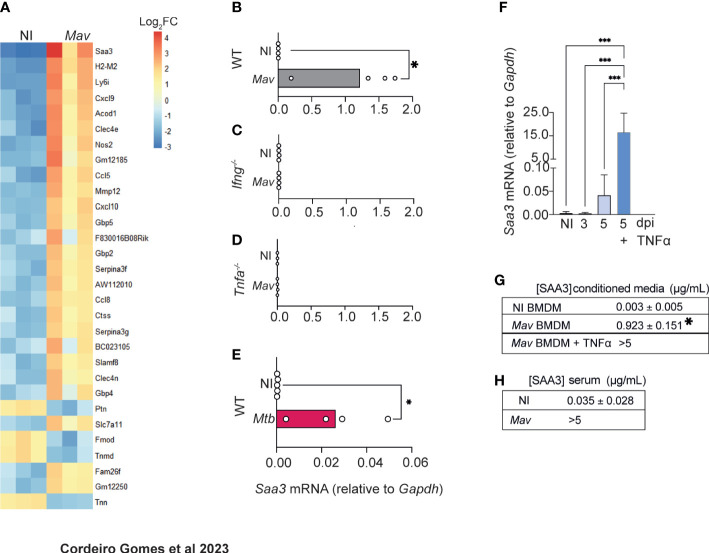
*M. avium*-infected macrophages produce SAA3. **(A)** Heatmap representing the 30 most differentially expressed genes in the femurs of *M. avium*-infected mice (*Mav*) compared with non-infected controls (NI). **(B–D)** *Saa3* expression relative to *Gapdh* in the femurs of non-infected (white bars) and *M. avium*-infected (gray bars) wild-type (WT, B), *Ifng*^-/-^ **(C)**, and *Tnfa*^-/-^ **(D)** mice. Bars indicate mean and circles depict individual mice analyzed. **(E)** *Saa3* expression in the bone marrow of mice infected with *M. tuberculosis* by the aerosol route. Bars indicate the average and circles depict each individual mouse. *, p<0.0,5, by Student’s *t* test. **(F)** *Saa3* expression relative to *Gapdh* in bone marrow derived macrophages. Bars represent the average ± standard deviation of the 4 replicates in each experimental condition. **(G)** Quantification of SAA3 in conditioned media from bone marrow derived macrophages, expressed as average ± standard deviation of the experimental condition. Results are pooled from 3 independent experiments. *, p<0.05, ***, p<0.001 by two-way ANOVA. **(H)** Measurement of SAA3 in the serum of *M. avium* -infected mice (*Mav*) compared to non-infected controls (NI) expressed as average ± standard deviation of the experimental group. N= 5 mice per group.

In the search for factors other than TNFα involved in increased osteogenesis, we noted that the most differentially upregulated gene in the bones of *M. avium*-infected mice was *Saa3* (**Figure 4A**). This gene codes for the Serum Amyloid A (SAA)3 protein, which has been recently implicated in bone homeostasis (32–35), namely by enhancing osteoclastogenesis (34). Quantitative real-time PCR analysis confirmed that the expression of *Saa3* is upregulated in the bone during *M. avium* infection (**Figure 4B**). Moreover, the expression of *Saa3* in the bone was dependent on the production of both IFNγ and TNFα, as mice deficient in either of these cytokines do not upregulate *Saa3* expression in the bone during *M. avium* infection (**Figures 4C, D**). Importantly, in mice infected with a low dose of *M. tuberculosis* by the aerosol route, the expression of *Saa3* in the bone marrow was also upregulated as compared with non-infected mice (**Figure 4E**).

We also found that *Saa3* was upregulated in BMDM 5 days after *M. avium* infection and was further augmented by the addition of TNFα to the macrophages’ cultures (**Figure 4F**). Corroborating the gene expression data, SAA3 was present in the conditioned media of infected macrophages, and the levels were further increased when infected macrophages were treated with TNFα (**Figure 4G**), as expected (36–39).

To determine whether the alterations in *Saa3* expression in the bone correlated with its systemic levels, SAA3 levels were measured in the serum of *M. avium*-infected and non-infected mice. Indeed, high concentrations of SAA3 were found in the serum of infected mice at 8 weeks post-infection (**Figure 4H**).

Thus far, our results suggest that during *M. avium* infection, the production of IFNγ leads to the activation of macrophages with the concomitant production of TNFα and SAA3. In the absence of TNFα, SAA3 was not expressed in the bone and the bone mass was not altered. Additionally, SAA3 was produced by TNFα-stimulated *M. avium*-infected macrophages.

### 3.5 SAA proteins directly impact bone turnover and are a potential biomarker of bone loss in patients with active Tuberculosis

**Figure 5:**
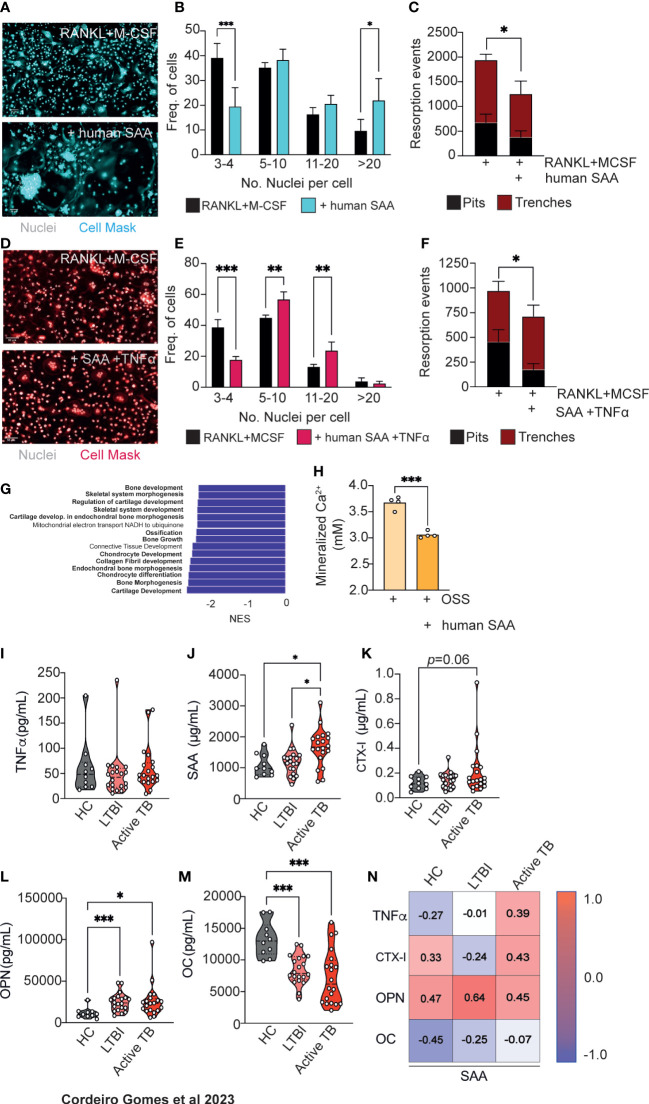
Human SAA increase osteoclastogenesis and decrease osteoblastogenesis and correlate with altered markers of bone turnover. **(A)** Osteoclast differentiation in the presence of saturating amounts of M-CSF and RANKL (top panel) and with the addition of human SAA (bottom panel). Scale bar: 50 μm **(B)** Frequency of multinucleated cells grouped by number of nuclei per cell. Data are pooled from three independent experiments. Bars represent the average ± standard deviation of the three independent experiments. **(C)** Enumeration of pits (black bars) and trenches (red bars). Data are representative of two independent experiments. Bars represent the average ± standard deviation of 4 replicates in each experimental group. **(D)** Osteoclast differentiation in the presence of saturating amounts of M-CSF and RANKL (left panel) and with the addition of human SAA and TNFα (right panel). Scale bar: 50 μm **(E)** Frequency of multinucleated cells grouped by number of nuclei per cell. Data are pooled from two independent experiments. Bars represent the average ± standard deviation of the two independent experiments. **(F)** Enumeration of pits (black bars) and trenches (red bars). Data are representative of two independent experiments. Bars represent the average ± standard deviation of 4 replicates in each experimental condition. *, p<0.0,5, ** p<0.01, ***, p<0.001 by Two-way ANOVA. **(G)** Top15 of most differently enriched pathways (from the GO Biological Process dataset) in the bones of infected mice compared to non-infected littermate controls. **(H)** Quantification of deposited calcium of murine bone marrow MSC cultured with osteoblast stimulatory supplement (OSS) plus 5 μg/mL human SAA. Bars indicate average; each circle depicts each of 4 replicates per experimental condition. Data are representative of two independent experiments. ***, p<0.001 by Student’s *t* test. **(I–M)** Measurement of TNFα **(I)**, SAA **(J)**, CTX-I **(K)**, Osteopontin (OPN, **(L)**, Osteocalcin (OC, **(M)** in the serum of healthy controls (HC), IGRA+ with latent tuberculosis infection (LTBI) individuals, and patients with active Tuberculosis (active TB). Violin plots depict the density, and each circle represents each patient. *, p<0.05, ***, p<0.001 by two-way ANOVA. **(N)** Pearson correlations between circulating levels of SAA and TNFα, or each of the different bone turnover markers.

We next decided to test whether SAA proteins directly impact bone turnover. SAA proteins are well-conserved in mammals (40) and have several isoforms (41). Although the human *Saa3* is a non-translated pseudogene (42), the human SAA1 and SAA2 display a remarkable homology to mouse SAA3 (34). To test if human SAA would also influence osteoclast differentiation, we used our previously described *in vitro* system incubating osteoclasts in the presence of human recombinant SAA1 and SAA2 proteins (human SAA). Osteoclastogenesis was increased (**Figures 5A, B**) but the number of resorption events was decreased (**Figure 5C**), indicating that besides SAA other soluble factors are important for the increased bone resorption (**Figures 3D–F**). According to our previous data, TNFα is a likely candidate (**Figures 3G–J**). Indeed, in osteoclasts cultured in the presence of TNFα and human SAA, we not only observed an increase of osteoclastogenesis, but also of osteoclast activity, measured as the proportion of trenches formed (**Figures 5D, F**). Overall, these results indicate that SAA and TNFα, which are produced by infected macrophages during mycobacteria infection, co-operate as pro-osteoclastogenic factors, which during mycobacteria infection are produced by infected macrophages.

Besides increasing osteoclastogenesis, SAA3 was also reported to inhibit osteoblastogenesis (32, 34, 35). Thus, we interrogated our transcriptomic data (**Figure 2A**) for possible differences in bone morphogenesis, bone development and growth, and ossification pathways and found that they were among the most differentially and statistically downregulated in the femurs of *M. avium*-infected mice compared with the femurs of littermate control mice (**Figure 5G**). This finding led to the hypothesis that osteoblastogenesis may also be impaired during mycobacterial infections. To test whether human SAA might also impact on osteoblastogenesis, MSC from the bone marrow of non-infected mice were isolated and osteoblast formation *in vitro* assays were performed in the presence and absence of human SAA. Osteoblast formation and bone matrix mineralization was detected by alizarin red staining. The addition of human SAA significantly reduced the calcium deposits in the cultures (**Figure 5H**).

The incidence of non-tuberculous mycobacterial infections, as *M. avium*, has been rising (43), namely in cystic fibrosis patients (44) and even in seemingly healthy children and adults (45). However, *M. tuberculosis* remains one of the deadliest human pathogens (11). Given the data suggesting a role for SAA in *M. avium* and *M. tuberculosis* mouse infection, as well as for human SAA in modulating osteoclast/osteoblast homeostasis, we went on to investigate whether the levels of SAA might change during active tuberculosis. For that, circulating levels of TNFα and SAA were measured in the plasma of a cohort of patients with active tuberculosis compared to those detected in healthy controls of unknown IGRA status or for IGRA+ (latent tuberculosis infection, LTBI) individuals. While TNFα serum levels were similar between the three groups (**Figure 5I**), patients with active tuberculosis showed higher systemic SAA levels as compared to both healthy and LTBI controls (**Figure 5J**). To correlate the alterations seen in SAA proteins to bone loss, we next measured the circulating levels of CTX-I and osteopontin (OPN) as a biomarkers of bone degradation, and of osteocalcin (OC) as a biomarker of bone formation (46), in the plasma of the study cohort. We found an increased amount of CTX-I in active tuberculosis patients which did not reach statistical significance (**Figure 5K**). The circulating levels of OPN was higher in the LTBI and active TB groups compared with healthy controls (**Figure 5L**). Bone formation was also impaired in LTBI and TB active groups as OC levels were reduced in these individuals compared to healthy controls (**Figure 5M**). Of note, there was a good correlation between the systemic levels of SAA, CTX-I, OPN and TNFα in patients with active TB (**Figure 5N**). These findings highlight the potential for the use of the systemic levels of SAA proteins as a biomarker of increased risk of bone loss during chronic mycobacterial infections.

## 4 Discussion

Although it is well established that mycobacteria can infect the bone (2) and that previous mycobacterial infection increases the risk of osteoporosis (4, 5), very little evidence of the pathophysiology of these alterations has been gathered. Our study shows that during chronic disseminated *M. avium* infection, IFNγ and TNFα induce bone loss, due to both increased resorption and decreased new bone formation. Our work advances the growing body of evidence supporting a reciprocal interaction between bone homeostasis and hematopoietic development. On one hand, alterations in LEPR+ MSC correlated with decreased bone formation. LEPR^+^ MSC were described as the major source of osteoblasts and adipocytes in the adult bone marrow (24) while they nurse hematopoietic maintenance and differentiation (17, 47), reinforcing the close relationship between hematopoiesis and the bone (3). In line with our observations, a previous study demonstrated that sepsis altered both hematopoietic stem cells and hematopoiesis-supporting MSC leading to a reduction in lymphocyte and osteoblast production (48). Previous reports have implied the bone marrow as a niche for mycobacteria (49, 50), even during intranasal infection (51). Besides macrophages, hematopoietic (52) and mesenchymal (53) stem cells are colonized by mycobacteria in the bone marrow, which may explain the hematopoietic (7) and bone dysfunctions. It was previously reported that mycobacterial infections skew the differentiation of hematopoietic stem cells towards the myeloid lineage (8–10). Here we further demonstrate that the numbers and the activity of osteoclasts are increased during infection. Overall, our findings suggest that even though higher numbers of monocytes and macrophages are required to fight off infection, a collateral damage is the exacerbated bone resorption.

The presence of mycobacteria in the bone has been traced back to thousands of years ago (2). However, instead of a direct colonization of bone cells, we found that the containment of mycobacteria inside macrophages and the ongoing immune response are responsible for the reduced bone mass observed. We also demonstrate that soluble factors produced by infected macrophages such as TNFα increased the number of trenches formed by osteoclasts. Contrary to the standard mode of bone resorption (the pit mode) the trenches result from a faster and longer resorption of bone as the osteoclasts move, creating deeper and parallel erosions on the bone surface (30, 31). Besides TNFα, we also found that M-CSF is upregulated in the bones of infected mice, in an IFNγ-dependent manner. We did not determine the source of this factor. Osteoblasts are an important source of M-CSF in the bone, and it is plausible that in response to the observed dysregulation in the bone mass during infection, osteoblasts increase the production of M-CSF. But we cannot exclude that other cells such as MSC and endothelial cells are also responsible for the increased production of M-CSF. This factor may contribute to attract monocytes and potentiate their differentiation into osteoclasts (54).

MSC are a key component of the hematopoietic niche. We found that MSC frequency is increased in the bone marrow parenchyma of infected mice. IFNγ and TNFα may impact MSC differentiation by various pathways. IFNγ has been shown to induce osteoblastogenesis and downregulate adipocyte differentiation (7, 55). However, during mycobacterial infection, IFNγ also induces TNFα production that, in turn, has been shown to repress osteoblastogenesis (56, 57) and increase osteoblast apoptosis (58). In contrast, a previous study showed that TNFα triggering of NF-kB fosters osteogenic differentiation in MSC at the expense of other lineages (59). Our transcriptomic analysis of the bone revealed that the gene pathways involved in MSC differentiation towards chondrocytes and osteoblasts were the most downregulated by infection, albeit explaining the increased frequency of MSC during infection. Additionally, morphometric analysis revealed a clear decrease in osteoblast numbers in infected mice. These data indicate that the combination of soluble factors released during mycobacterial infection results in decreased osteoblastogenesis.

Our study reveals that both IFNγ and TNFα are critically involved in the pathophysiology of bone loss during mycobacterial infection, which is consistent with their recognized effects on the hematopoiesis and the above-mentioned interconnections between hematopoiesis and bone homeostasis. Interestingly, it was demonstrated that patients with autosomal dominant IFNγR1 or STAT1 deficiency showed enhanced osteoclastogenesis (3), which could be contradictory with our findings. However, this observation may be explained by the variable penetrance of disease (60) and by the fact that T cells from these patients still produce TNFα upon stimulation (61), which could mediate the increased osteoclastogenesis. Furthermore, deficiency in STAT1 also disrupts the IFNα and β signaling pathways (62, 63) that in turn could enhance IL17 production, leading to increased osteoclast activation and bone resorption (64) as gain of function STAT1 mutations induce attenuated Th17 response (63). Of note, besides genetic alterations, the response to ongoing mycobacterial infection and even to its treatment is modulated by individual factors such as the microbiome constitution (65). Therefore, it is possible that these factors also influence bone homeostasis. The study of possible bone loss in these patients warrants further research and may shed light into alternative or complementary mechanisms to those reported here. It will be important to directly address the role of SAA proteins in bone loss using mouse models deficient for SAA1, SAA2 and SAA3. Furthermore, human studies to determine whether individual susceptibilities such as differences in the immune response shaped by the individual’s microbiome conditionate low bone mass will be very important to better translate these animal derived mechanisms to humans.

The study of gene expression in the bones of mice revealed that one of the genes which is most upregulated by mycobacterial infection is *Saa3*. This is in agreement with previous observations that *Saa3* expression is upregulated in bone marrow-derived macrophages and in the lungs after infection with *M. tuberculosis* (50, 52). SAA proteins are described as acute phase proteins that are expressed mostly by hepatic macrophages in the liver (36) but also by monocytes and macrophages in other tissues, chondrocytes, adipocytes, and osteoblasts in response to inflammatory stimuli (66–75). More recently, SAA were shown to have both catabolic and anti-anabolic roles in bone turnover (32–35). Here, we showed that SAA proteins are produced by macrophages after infection with mycobacteria and that the levels of these proteins are significantly increased in circulation, both in experimental models of infection with mycobacteria and in humans with LTBI or active TB. We experimentally confirmed that SAA, together with inflammatory cytokines, directly contributes to increased bone degradation and decreased bone formation (**Figure 5**). Additionally, we found evidence that SAA proteins strongly associate with bone alterations in both *M. avium* and *M. tuberculosis* infections, being also altered in the serum of TB patients.

Biochemical markers of bone turnover such as CTX-I, OPN and OC provide a non-invasive tool for the clinical assessment and for guiding and monitoring of bone loss treatment (46). Nevertheless, some studies have reported that despite no alteration in bone turnover markers levels, alterations in the microstructure and mineral density may be already observed by high resolution peripheral computed tomography (HR-pQCT) and dual X- ray absorptiometry (DXA), respectively (76–78), in the absence of alterations in bone turnover markers. As DXA and HR-pQCT are not routinely performed on tuberculosis patients, relying on CTX-I, OPN and OC in the serum may underestimate the impact of infection on the bone health of these individuals. Our results are the first evidence correlating the circulating levels of SAA proteins and bone turnover markers in humans with active tuberculosis. A prospective study to correlate the circulating SAA levels and the bone mineral density in a cohort of tuberculosis patients is warranted, to assess the utility of SAA as a new bone loss marker.

Furthermore, as we show that SAA production and bone loss are dependent on inflammatory cytokines, the clinical relevance of SAA proteins as a biomarker of bone loss may be extended to other pathologies such as chronic cardiovascular diseases (79, 80), inflammatory rheumatic diseases as well as COVID-19 (81), in which the systemic levels of SAA are elevated. These results lay the ground for further studies to contribute to a deeper understanding of the mechanisms underlying bone loss during inflammatory diseases and consequently to the proposal of new therapeutic strategies to prevent or cure these disorders.

## Data availability statement

The datasets presented in this study can be found in online repositories. The names of the repository/repositories and accession number(s) can be found below: https://www.ncbi.nlm.nih.gov/geo/, GSE215856.

## Ethics statement

The studies involving human participants were reviewed and approved by Portuguese Comissão de Ética para a Saúde da ARS Norte (#T792). The patients/participants provided their written informed consent to participate in this study. The animal study was reviewed and approved by i3S Ethical committee.

## Author contributions

AG, ML and MG supervised the study. AG conceptualized, designed and performed the experiments. AG, MS and MG wrote the manuscript. DS conceptualized and performed some experiments. TO, OF, DS, AM and TS performed experiments. AF, MT and MS provided human samples. AG, RP and LP performed the transcriptomic analysis. All authors contributed to the article and approved the submitted version.

## References

[1] Redlich K Smolen JS. Inflammatory bone loss: pathogenesis and therapeutic intervention. Nat Rev Drug Discovery (2012) 11(3):234–50. doi: 10.1038/nrd3669 22378270

[2] Hogan JI Hurtado RM Nelson SB. Mycobacterial musculoskeletal infections. Thorac Surg Clin (2019) 29(1):85–94. doi: 10.1016/j.thorsurg.2018.09.007 30454925

[3] Tsumura M Miki M Mizoguchi Y Hirata O Nishimura S Tamaura M. Enhanced osteoclastogenesis in patients with MSMD due to impaired response to IFN-gamma. J Allergy Clin Immunol (2022) 149(1):252–61 e6. doi: 10.1016/j.jaci.2021.05.018 34176646

[4] Yeh JJ Wang YC Lin CC Lin CL Hsu WH. Association of respiratory tuberculosis with incident bone fracture: bridging the tuberculosis airway infection and the osteoporotic bone. PloS One (2016) 11(12):e0168673. doi: 10.1371/journal.pone.0168673 28005963 PMC5178997

[5] Choi CJ Choi WS Kim CM Lee SY Kim KS. Risk of sarcopenia and osteoporosis in Male tuberculosis survivors: Korea national health and nutrition examination survey. Sci Rep (2017) 7(1):13127. doi: 10.1038/s41598-017-12419-y 29030560 PMC5640648

[6] Oliveira TC Gomes MS Gomes AC. The crossroads between infection and bone loss. Microorganisms (2020) 8(11) 1765–82. doi: 10.3390/microorganisms8111765 PMC7698271 33182721

[7] Gomes AC Saraiva M Gomes MS. The bone marrow hematopoietic niche and its adaptation to infection. Semin Cell Dev Biol (2021) 112:37–48. doi: 10.1016/j.semcdb.2020.05.014 32553581

[8] Baldridge MT King KY Boles NC Weksberg DC Goodell MA. Quiescent haematopoietic stem cells are activated by IFN-gamma in response to chronic infection. Nature (2010) 465(7299):793–7. doi: 10.1038/nature09135 PMC2935898 20535209

[9] Matatall KA Jeong M Chen S Sun D Chen F Mo Q. Chronic infection depletes hematopoietic stem cells through stress-induced terminal differentiation. Cell Rep (2016) 17(10):2584–95. doi: 10.1016/j.celrep.2016.11.031 PMC5161248 27926863

[10] Matatall KA Shen CC Challen GA King KY. Type II interferon promotes differentiation of myeloid-biased hematopoietic stem cells. Stem Cells (2014) 32(11):3023–30. doi: 10.1002/stem.1799 PMC4198460 25078851

[11] Global tuberculosis report 2021. Geneva: World Health Organization; (2021). Licence: CC BY-NC-SA 3.0 IGO.

[12] Bouxsein ML Boyd SK Christiansen BA Guldberg RE Jepsen KJ Muller R. Guidelines for assessment of bone microstructure in rodents using micro-computed tomography. J Bone Miner Res (2010) 25(7):1468–86. doi: 10.1002/jbmr.141 20533309

[13] Sousa DM Baldock PA Enriquez RF Zhang L Sainsbury A Lamghari M. Neuropeptide y Y1 receptor antagonism increases bone mass in mice. Bone (2012) 51(1):8–16. doi: 10.1016/j.bone.2012.03.020 22484690

[14] van Gaalen SM Kruyt MC Geuze RE de Bruijn JD Alblas J Dhert WJ. Use of fluorochrome labels in *in vivo* bone tissue engineering research. Tissue Eng Part B Rev (2010) 16(2):209–17. doi: 10.1089/ten.teb.2009.0503 19857045

[15] Kawamoto T Kawamoto K. Preparation of thin frozen sections from nonfixed and undecalcified hard tissues using kawamoto’s film method (2020). Methods Mol Biol (2021) 2230:259–81. doi: 10.1007/978-1-0716-1028-2_15 33197019

[16] Chow A Lucas D Hidalgo A Mendez-Ferrer S Hashimoto D Scheiermann C. Bone marrow CD169+ macrophages promote the retention of hematopoietic stem and progenitor cells in the mesenchymal stem cell niche. J Exp Med (2011) 208(2):261–71. doi: 10.1084/jem.20101688 PMC3039855 21282381

[17] Cordeiro Gomes A Hara T Lim VY Herndler-Brandstetter D Nevius E Sugiyama T. Hematopoietic stem cell niches produce lineage-instructive signals to control multipotent progenitor differentiation. Immunity (2016) 45(6):1219–31. doi: 10.1016/j.immuni.2016.11.004 PMC5538583 27913094

[18] Love MI Huber W Anders S. Moderated estimation of fold change and dispersion for RNA-seq data with DESeq2. Genome Biol (2014) 15(12):550. doi: 10.1186/s13059-014-0550-8 25516281 PMC4302049

[19] Bult CJ Blake JA Smith CL Kadin JA Richardson JE Mouse Genome Database G. Mouse genome database (MGD) 2019. Nucleic Acids Res (2019) 47(D1):D801–D6. doi: 10.1093/nar/gky1056 PMC6323923 30407599

[20] Shannon P Markiel A Ozier O Baliga NS Wang JT Ramage D. Cytoscape: a software environment for integrated models of biomolecular interaction networks. Genome Res (2003) 13(11):2498–504. doi: 10.1101/gr.1239303 PMC403769 14597658

[21] Merico D Isserlin R Stueker O Emili A Bader GD. Enrichment map: a network-based method for gene-set enrichment visualization and interpretation. PloS One (2010) 5(11):e13984. doi: 10.1371/journal.pone.0013984 21085593 PMC2981572

[22] Nevius E Pinho F Dhodapkar M Jin H Nadrah K Horowitz MC. Oxysterols and EBI2 promote osteoclast precursor migration to bone surfaces and regulate bone mass homeostasis. J Exp Med (2015) 212(11):1931–46. doi: 10.1084/jem.20150088 PMC4612084 26438360

[23] Gomes AC Moreira AC Silva T Neves JV Mesquita G Almeida AA. IFN-gamma-Dependent reduction of erythrocyte life span leads to anemia during mycobacterial infection. J Immunol (2019) 203(9):2485–96. doi: 10.4049/jimmunol.1900382 31562210

[24] Zhou BO Yue R Murphy MM Peyer JG Morrison SJ. Leptin-receptor-expressing mesenchymal stromal cells represent the main source of bone formed by adult bone marrow. Cell Stem Cell (2014) 15(2):154–68. doi: 10.1016/j.stem.2014.06.008 PMC4127103 24953181

[25] Abu-Amer Y. NF-κB signaling and bone resorption. Osteoporos Int (2013) 24(9):2377–86. doi: 10.1007/s00198-013-2313-x PMC3884829 23468073

[26] Nevius E Gomes AC Pereira JP. Inflammatory cell migration in rheumatoid arthritis: a comprehensive review. Clin Rev Allergy Immunol (2016) 51(1):59–78. doi: 10.1007/s12016-015-8520-9 26511861 PMC5785098

[27] Amarasekara DS Yun H Kim S Lee N Kim H Rho J. Regulation of osteoclast differentiation by cytokine networks. Immune Netw (2018) 18(1):e8. doi: 10.4110/in.2018.18.e8 29503739 PMC5833125

[28] Gao Y Grassi F Ryan MR Terauchi M Page K Yang X. IFN-gamma stimulates osteoclast formation and bone loss *in vivo via* antigen-driven T cell activation. J Clin Invest. (2007) 117(1):122–32. doi: 10.1172/JCI30074 PMC1697800 17173138

[29] Appelberg R Castro AG Pedrosa J Silva RA Orme IM Minoprio P. Role of gamma interferon and tumor necrosis factor alpha during T-cell-independent and -dependent phases of mycobacterium avium infection. Infect Immun (1994) 62(9):3962–71. doi: 10.1128/iai.62.9.3962-3971.1994 PMC303054 8063414

[30] Soe K Delaisse JM. Time-lapse reveals that osteoclasts can move across the bone surface while resorbing. J Cell Sci (2017) 130(12):2026–35. doi: 10.1242/jcs.202036 PMC5482982 28473470

[31] Merrild DM Pirapaharan DC Andreasen CM Kjaersgaard-Andersen P Moller AM Ding M. Pit- and trench-forming osteoclasts: a distinction that matters. Bone Res (2015) 3:15032. doi: 10.1038/boneres.2015.32 26664853 PMC4665108

[32] Choudhary S Goetjen A Estus T Jacome-Galarza CE Aguila HL Lorenzo J. Serum amyloid A3 secreted by preosteoclasts inhibits parathyroid hormone-stimulated cAMP signaling in murine osteoblasts. J Biol Chem (2016) 291(8):3882–94. doi: 10.1074/jbc.M115.686576 PMC4759168 26703472

[33] Choudhary S Santone E Yee S-P Lorenzo J Adams DJ Goetjen A. Continuous PTH in Male mice causes bone loss because it induces serum amyloid a. Endocrinology (2018) 159(7):2759–76. doi: 10.1210/en.2018-00265 PMC6692876 29757436

[34] Thaler R Sturmlechner I Spitzer S Riester SM Rumpler M Zwerina J. Acute-phase protein serum amyloid A3 is a novel paracrine coupling factor that controls bone homeostasis. FASEB J (2015) 29(4):1344–59. doi: 10.1096/fj.14-265512 PMC4396610 25491310

[35] Thaler R Zwerina J Rumpler M Spitzer S Gamsjaeger S Paschalis EP. Homocysteine induces serum amyloid A3 in osteoblasts *via* unlocking RGD-motifs in collagen. FASEB J (2013) 27(2):446–63. doi: 10.1096/fj.12-208058 23085993

[36] Sack GH Jr. Serum amyloid a - a review. Mol Med (2018) 24(1):46. doi: 10.1186/s10020-018-0047-0 30165816 PMC6117975

[37] Sommer G Weise S Kralisch S Scherer PE Lossner U Bluher M. The adipokine SAA3 is induced by interleukin-1beta in mouse adipocytes. J Cell Biochem (2008) 104(6):2241–7. doi: 10.1002/jcb.21782 18452164

[38] Maier W Altwegg LA Corti R Gay S Hersberger M Maly FE. Inflammatory markers at the site of ruptured plaque in acute myocardial infarction: locally increased interleukin-6 and serum amyloid a but decreased c-reactive protein. Circulation (2005) 111(11):1355–61. doi: 10.1161/01.CIR.0000158479.58589.0A 15753219

[39] Betts JC Cheshire JK Akira S Kishimoto T Woo P. The role of NF-kappa b and NF-IL6 transactivating factors in the synergistic activation of human serum amyloid a gene expression by interleukin-1 and interleukin-6. J Biol Chem (1993) 268(34):25624–31. doi: 10.1016/S0021-9258(19)74435-4 8244997

[40] Uhlar CM Burgess CJ Sharp PM Whitehead AS. Evolution of the serum amyloid a (SAA) protein superfamily. Genomics (1994) 19(2):228–35. doi: 10.1006/geno.1994.1052 8188253

[41] Uhlar CM Whitehead AS. The major vertebrate acute-phase reactant. Eur J Biochem (1999) 265(2):501–23. doi: 10.1046/j.1432-1327.1999.00657.x 10504381

[42] Kluve-Beckerman B Drumm ML Benson MD. Nonexpression of the human serum amyloid a three (SAA3) gene. DNA Cell Biol (1991) 10(9):651–61. doi: 10.1089/dna.1991.10.651 1755958

[43] Donohue MJ Wymer L. Increasing prevalence rate of nontuberculous mycobacteria infections in five states, 2008-2013. Ann Am Thorac Soc (2016) 13(12):2143–50. doi: 10.1513/AnnalsATS.201605-353OC 27681202

[44] Viviani L Harrison MJ Zolin A Haworth CS Floto RA. Epidemiology of nontuberculous mycobacteria (NTM) amongst individuals with cystic fibrosis (CF). J Cyst Fibros. (2016) 15(5):619–23. doi: 10.1016/j.jcf.2016.03.002 27050794

[45] Ratnatunga CN Lutzky VP Kupz A Doolan DL Reid DW Field M. The rise of non-tuberculosis mycobacterial lung disease. Front Immunol (2020) 11:303. doi: 10.3389/fimmu.2020.00303 32194556 PMC7062685

[46] Naylor K Eastell R. Bone turnover markers: use in osteoporosis. Nat Rev Rheumatol (2012) 8(7):379–89. doi: 10.1038/nrrheum.2012.86 22664836

[47] Miao R Chun H Feng X Gomes AC Choi J Pereira JP. Competition between hematopoietic stem and progenitor cells controls hematopoietic stem cell compartment size. Nat Commun (2022) 13(1):4611. doi: 10.1038/s41467-022-32228-w 35941168 PMC9360400

[48] Terashima A Okamoto K Nakashima T Akira S Ikuta K Takayanagi H. Sepsis-induced osteoblast ablation causes immunodeficiency. Immunity (2016) 44(6):1434–43. doi: 10.1016/j.immuni.2016.05.012 27317262

[49] Beamer G Major S Das B Campos-Neto A. Bone marrow mesenchymal stem cells provide an antibiotic-protective niche for persistent viable mycobacterium tuberculosis that survive antibiotic treatment. Am J Pathol (2014) 184(12):3170–5. doi: 10.1016/j.ajpath.2014.08.024 PMC4261085 25451154

[50] Mayito J Andia I Belay M Jolliffe DA Kateete DP Reece ST. Anatomic and cellular niches for mycobacterium tuberculosis in latent tuberculosis infection. J Infect Dis (2019) 219(5):685–94. doi: 10.1093/infdis/jiy579 PMC6376907 30376080

[51] Kager LM Runge JH Nederveen AJ Roelofs JJ Stoker J Maas M. A new murine model to study musculoskeletal tuberculosis (short communication). Tuberculosis (Edinb). (2014) 94(3):306–10. doi: 10.1016/j.tube.2014.01.002 24572169

[52] Tornack J Reece ST Bauer WM Vogelzang A Bandermann S Zedler U. Human and mouse hematopoietic stem cells are a depot for dormant mycobacterium tuberculosis. PloS One (2017) 12(1):e0169119. doi: 10.1371/journal.pone.0169119 28046053 PMC5207496

[53] Das B Kashino SS Pulu I Kalita D Swami V Yeger H. CD271(+) bone marrow mesenchymal stem cells may provide a niche for dormant mycobacterium tuberculosis. Sci Transl Med (2013) 5(170):170ra13. doi: 10.1126/scitranslmed.3004912 PMC3616630 23363977

[54] Emoto T Lu J Sivasubramaniyam T Maan H Khan AB Abow AA. Colony stimulating factor-1 producing endothelial cells and mesenchymal stromal cells maintain monocytes within a perivascular bone marrow niche. Immunity (2022) 55(5):862–78 e8. doi: 10.1016/j.immuni.2022.04.005 35508166

[55] Tang M Tian L Luo G Yu X. Interferon-Gamma-Mediated osteoimmunology. Front Immunol (2018) 9:1508. doi: 10.3389/fimmu.2018.01508 30008722 PMC6033972

[56] Yang N Wang G Hu C Shi Y Liao L Shi S. Tumor necrosis factor α suppresses the mesenchymal stem cell osteogenesis promoter miR-21 in estrogen deficiency–induced osteoporosis. J Bone Mineral Res (2013) 28(3):559–73. doi: 10.1002/jbmr.1798 23074166

[57] Zhao L Huang J Zhang H Wang Y Matesic LE Takahata M. Tumor necrosis factor inhibits mesenchymal stem cell differentiation into osteoblasts *via* the ubiquitin E3 ligase Wwp1. Stem Cells (2011) 29(10):1601–10. doi: 10.1002/stem.703 PMC3708970 21809421

[58] Fischer V Haffner-Luntzer M. Interaction between bone and immune cells: implications for postmenopausal osteoporosis. Semin Cell Dev Biol (2022) 123:14–21. doi: 10.1016/j.semcdb.2021.05.014 34024716

[59] Hess K Ushmorov A Fiedler J Brenner RE Wirth T. TNFα promotes osteogenic differentiation of human mesenchymal stem cells by triggering the NF-κB signaling pathway. Bone (2009) 45(2):367–76. doi: 10.1016/j.bone.2009.04.252 19414075

[60] Gruber C Bogunovic D. Incomplete penetrance in primary immunodeficiency: a skeleton in the closet. Hum Genet (2020) 139(6-7):745–57. doi: 10.1007/s00439-020-02131-9 PMC7275875 32067110

[61] Kerner G Rosain J Guerin A Al-Khabaz A Oleaga-Quintas C Rapaport F. Inherited human IFN-gamma deficiency underlies mycobacterial disease. J Clin Invest. (2020) 130(6):3158–71. doi: 10.1172/JCI135460 PMC7260033 32163377

[62] Dupuis S Jouanguy E Al-Hajjar S Fieschi C Al-Mohsen IZ Al-Jumaah S. Impaired response to interferon-alpha/beta and lethal viral disease in human STAT1 deficiency. Nat Genet (2003) 33(3):388–91. doi: 10.1038/ng1097 12590259

[63] Liu L Okada S Kong XF Kreins AY Cypowyj S Abhyankar A. Gain-of-function human STAT1 mutations impair IL-17 immunity and underlie chronic mucocutaneous candidiasis. J Exp Med (2011) 208(8):1635–48. doi: 10.1084/jem.20110958 PMC3149226 21727188

[64] Le Goff B Bouvard B Lequerre T Lespessailles E Marotte H Pers YM. Implication of IL-17 in bone loss and structural damage in inflammatory rheumatic diseases. Mediators Inflamm (2019) 2019:8659302. doi: 10.1155/2019/8659302 31485194 PMC6710740

[65] Namasivayam S Sher A Glickman MS Wipperman MF. The microbiome and tuberculosis: early evidence for cross talk. mBio (2018) 9(5):e01420–18. doi: 10.1128/mBio.01420-18 PMC6143735 30228238

[66] Vallon R Freuler F Desta-Tsedu N Robeva A Dawson J Wenner P. (apoSAA) expression is up-regulated in rheumatoid arthritis and induces transcription of matrix metalloproteinases. J Immunol (2001) 166(4):2801–7. doi: 10.4049/jimmunol.166.4.2801 11160347

[67] Malle E De Beer FC. Human serum amyloid a (SAA) protein: a prominent acute-phase reactant for clinical practice. Eur J Clin Invest. (1996) 26(6):427–35. doi: 10.1046/j.1365-2362.1996.159291.x 8817153

[68] Meek RL Eriksen N Benditt EP. Murine serum amyloid A3 is a high density apolipoprotein and is secreted by macrophages. Proc Natl Acad Sci U S A. (1992) 89(17):7949–52. doi: 10.1073/pnas.89.17.7949 PMC49832 1518819

[69] Kumon Y Loose LD Birbara CA Sipe JD. Rheumatoid arthritis exhibits reduced acute phase and enhanced constitutive serum amyloid a protein in synovial fluid relative to serum. a comparison with c-reactive protein. J Rheumatol (1997) 24(1):14–9. 9002005

[70] Meek RL Benditt EP. Amyloid a gene family expression in different mouse tissues. J Exp Med (1986) 164(6):2006–17. doi: 10.1084/jem.164.6.2006 PMC2188489 3783088

[71] Thorn CF Lu ZY Whitehead AS. Tissue-specific regulation of the human acute-phase serum amyloid a genes, SAA1 and SAA2, by glucocorticoids in hepatic and epithelial cells. Eur J Immunol (2003) 33(9):2630–9. doi: 10.1002/eji.200323985 12938239

[72] Zerega B Pagano A Pianezzi A Ulivi V Camardella L Cancedda R. Expression of serum amyloid a in chondrocytes and myoblasts differentiation and inflammation: possible role in cholesterol homeostasis. Matrix Biol (2004) 23(1):35–46. doi: 10.1016/j.matbio.2004.02.002 15172036

[73] Reigstad CS Lunden GO Felin J Backhed F. Regulation of serum amyloid A3 (SAA3) in mouse colonic epithelium and adipose tissue by the intestinal microbiota. PloS One (2009) 4(6):e5842. doi: 10.1371/journal.pone.0005842 19513118 PMC2688757

[74] Yamada T Wada A Itoh K Igari J. Serum amyloid a secretion from monocytic leukaemia cell line THP-1 and cultured human peripheral monocytes. Scand J Immunol (2000) 52(1):7–12. doi: 10.1046/j.1365-3083.2000.00734.x 10886778

[75] Han CY Subramanian S Chan CK Omer M Chiba T Wight TN. Adipocyte-derived serum amyloid A3 and hyaluronan play a role in monocyte recruitment and adhesion. Diabetes (2007) 56(9):2260–73. doi: 10.2337/db07-0218 17563062

[76] Gensburger D Boutroy S Chapurlat R Nove-Josserand R Roche S Rabilloud M. Reduced bone volumetric density and weak correlation between infection and bone markers in cystic fibrosis adult patients. Osteoporos Int (2016) 27(9):2803–13. doi: 10.1007/s00198-016-3612-9 27165286

[77] Mathiesen IH Hitz MF Katzenstein TL Oturai P Skov M Jorgensen NR. Markers of bone turnover are reduced in patients with CF related diabetes; the role of glucose. J Cyst Fibros. (2019) 18(3):436–41. doi: 10.1016/j.jcf.2019.01.004 30928333

[78] Putman MS Milliren CE Derrico N Uluer A Sicilian L Lapey A. Compromised bone microarchitecture and estimated bone strength in young adults with cystic fibrosis. J Clin Endocrinol Metab (2014) 99(9):3399–407. doi: 10.1210/jc.2014-1982 PMC4154107 24926955

[79] Katayama T Nakashima H Honda Y Suzuki S Yamamoto T Iwasaki Y. The relationship between acute phase serum amyloid a (SAA) protein concentrations and left ventricular systolic function in acute myocardial infarction patients treated with primary coronary angioplasty. Int Heart J (2007) 48(1):45–55. doi: 10.1536/ihj.48.45 17379978

[80] Johnson BD Kip KE Marroquin OC Ridker PM Kelsey SF Shaw LJ. Serum amyloid a as a predictor of coronary artery disease and cardiovascular outcome in women: the national heart, lung, and blood institute-sponsored women’s ischemia syndrome evaluation (WISE). Circulation (2004) 109(6):726–32. doi: 10.1161/01.CIR.0000115516.54550.B1 14970107

[81] Soric Hosman I Kos I Lamot L. Serum amyloid a in inflammatory rheumatic diseases: a compendious review of a renowned biomarker. Front Immunol (2020) 11:631299. doi: 10.3389/fimmu.2020.631299 33679725 PMC7933664

