# Supplementary material for: Serum amyloid A proteins reduce bone mass during mycobacterial infections
Source: Front Immunol. 2023 Apr 21;14:1168607. doi: 10.3389/fimmu.2023.1168607 (PMC10161249; doi:10.3389/fimmu.2023.1168607)
Supplement: Supplementary file 1 [file DataSheet_1.pdf]

frontiers

Supplementary Material

Serum Amyloid A proteins reduce bone mass during mycobacterial infections

Ana Cordeiro Gomes*, Daniela Monteiro Sousa, Tiago Carvalho Oliveira, Óscar Fonseca, Ricardo J. Pinto, Diogo Silvério, Ana Isabel Fernandes, Ana C. Moreira, Tânia Silva, Maria José Teles, Luísa Pereira, Margarida Saraiva, Meriem Lamghari, Maria Salomé Gomes

*** Correspondence:** Ana Cordeiro Gomes: ana.c.gomes@i3s.up.pt

1 Supplementary Figures and Tables

1.1 Supplementary Figures

Supplementary Table 1. Demographics of human subjects enrolled in the study.

	Healthy controls (n=10)	LTBI patients (n=20)	Active T
Age	49±7	54±19	50±17
Sex<br>No. males<br>No. females	 6<br>4	 9<br>11	 9<br>11
IGRA status	Unknown	Positive	Positive
Sputum smear and culture results	Unknown	Negative	Positive
Symptoms or clinical signs (including thorax imaging) of active Tuberculosis	No	No	Yes

Supplementary Table 2. Sequences of primers used in qRT-PCR.

Gene	Forward Primer Sequence	Reverse Primer Sequence
Acp5	5’- GCTGGAAACCATCATCACCT - 3’	5’- TGAAGCGCAAACGGTAGT - 3’
Csf1	5’- GGGCCTCCTGTTCTACAAGT - 3’	5’- AGGAGAGGGTAGTGGTGGAT - 3’
Csf1r	5'-TTGGACTGGCTAGGGACATC-3’	5'-GGTTCAGACCAAGCGAGAAG-3’
Rank	5’- GTGCTGCTGGTTCCACTG - 3’	5’- CCGTCCGAGATGCTCATAAT- 3’
Saa3	5'-ACATGTGGCGAGCCTACTCT-3'	5'-GAGTCCTCTGCTCCATGTCC-3’
Gapdh	5’- TGTGTCCGTCGTGGATCTGA - 3’	5’- CCTGCTTCACCACCTTCTTGA - 3’

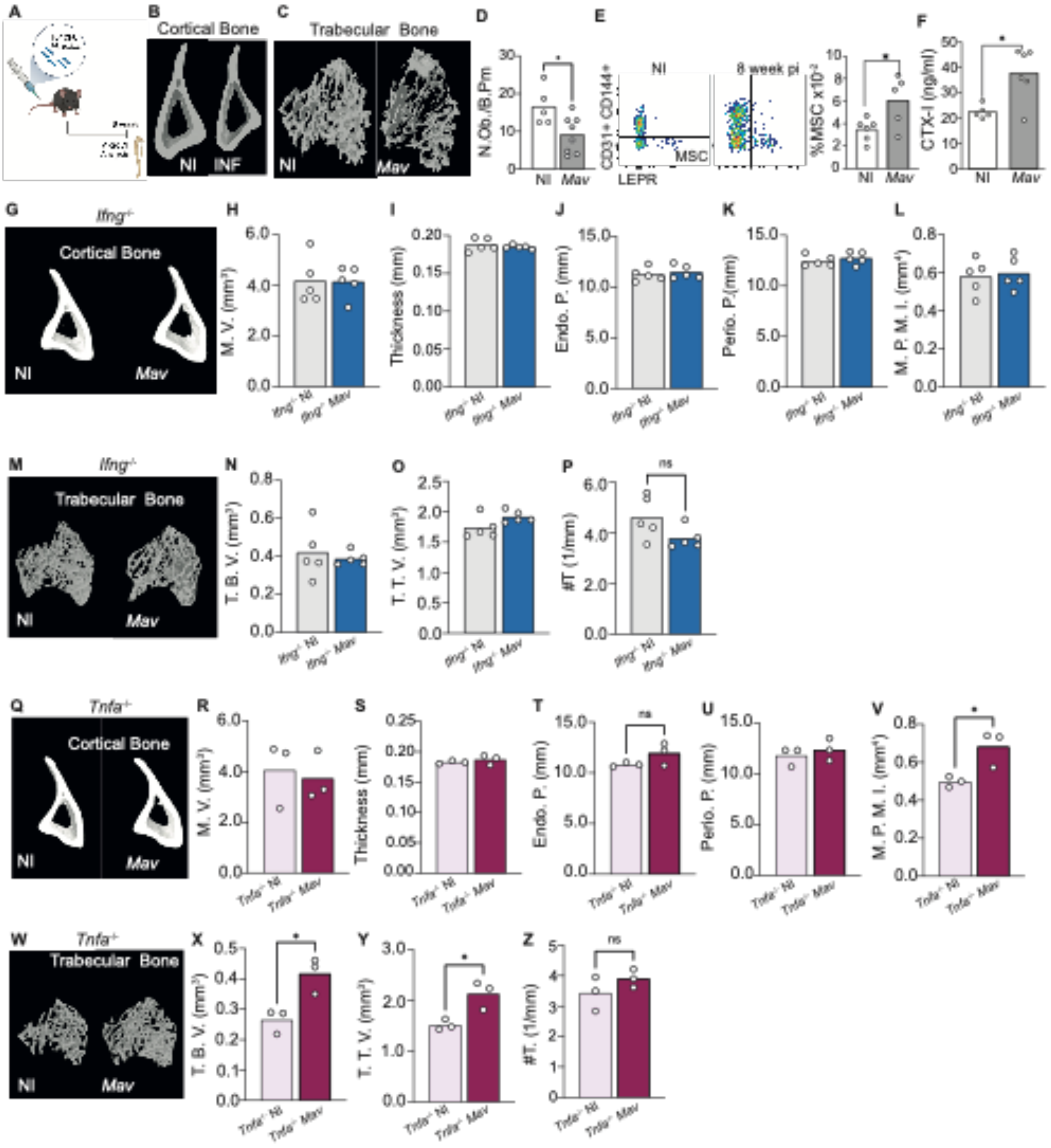

Supplementary Figure 1. Cordeiro Gomes et al 2023

Supplementary Figure 1. ***Mycobacterium avium*** **infection increases bone degradation and reduces bone formation. A)** Representative scheme of in vivo *M. avium* infection. **B-C)** Representative image of the cortical (**B**) and trabecular (**C**) tibial bone of *M. avium*-infected (*Mav* bars) and non-infected mice (NI bars) scanned by micro-CT. **D)** Enumeration of the osteoblast number per bone perimeter (N.Ob./B.Pm.) in infected mice (*Mav* bars) *vs* non-infected controls (NI bars). **E)** Enumeration of the frequency of LEPR+ MSC in the bone marrow of infected and non-infected mice by flow cytometry. **F)** Quantification of CTX-I in serum. N=5-8 mice per experimental group; data representative of two independent experiments. Bars indicate average and each dot represents each mouse. **G**) Representative image of the cortical tibial bone of *M. avium*-infected (*Mav* bars) and non-infected (NI bars) *Ifng*$^{-/-}$ mice scanned by micro-CT. **H-L)** Measurement of the marrow volume (M. V., **A**), cortical thickness (**I**), endocortical perimeter (Endo. P., **J**), periosteal perimeter (Perio. P., **K**), and mean polar moment of inertia (M. P. M. I., **L**) in *M. avium*-infected *Infgamma*$^{-/-}$ mice (*Mav* bars) compared to non-infected littermate controls (NI bars). **M)** Representative image of the trabecular tibial bone of *M. avium*-infected (*Mav* bars) and non-infected (NI bars) *Ifng*$^{-/-}$ mice scanned by micro-CT. **N-P**) Measurement of the trabecular bone volume (T. B. V., **N**), trabecular tissue volume (T. T. V., **N**), trabecular number (#T, **P**) in *M. avium*-infected *Infg*$^{-/-}$ mice (*Mav* bars) and non-infected controls (NI bars). **Q)** Representative image of the cortical tibial bone of infected and non-infected *Tnfa*$^{-/-}$ mice scanned by micro-CT. **R-V)** Measurement of the marrow volume (M. V., **R**), cortical thickness (**S**), endocortical perimeter (Endo. P., **T**), periosteal perimeter (Perio. P., **U**), and mean polar moment of inertia (M. P. M. I., **V**) in *M. avium*-infected *Tnfa*$^{-/-}$ mice (*Mav* bars) compared to non-infected littermate controls (NI bars). **W**) Representative image of the trabecular tibial bone of infected and non-infected *Tnfa*$^{-/-}$ mice scanned by micro-CT. **X-Z**) Measurement of the trabecular bone volume (T. B.V., **X**), trabecular tissue volume (T.T.V., **Y**), trabecular number (#T, **Z**) in *M. avium*-infected *Tnfa*$^{-/-}$ mice (*Mav* bars) and non-infected controls (NI bars). N=3-5 mice per experimental group; data representative of two independent experiments. Bars indicate average; each dot depicts each individual mouse analyzed *, $p<0.05$, ** $p<0.01$, ***, $p<0.001$ by Student's *t* test.

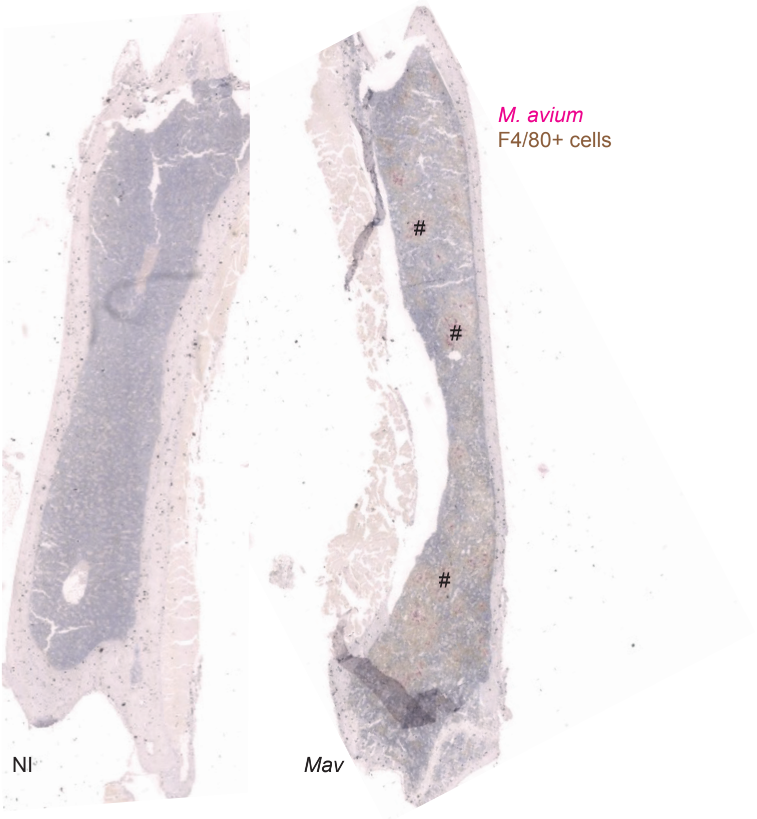

Supplementary Figure 2. In infected bones, *M. avium* resides mostly inside macrophages distributed throughout the bone marrow parenchyma. 5-µm thick sections of non-infected (NI, left panel) and infected (*Mav*, right panel) femurs stained with anti-F4/80 and Ziehl-Neelsen. (#) mark F4/80+ (brown) cells infected with *M. avium* (red rods).

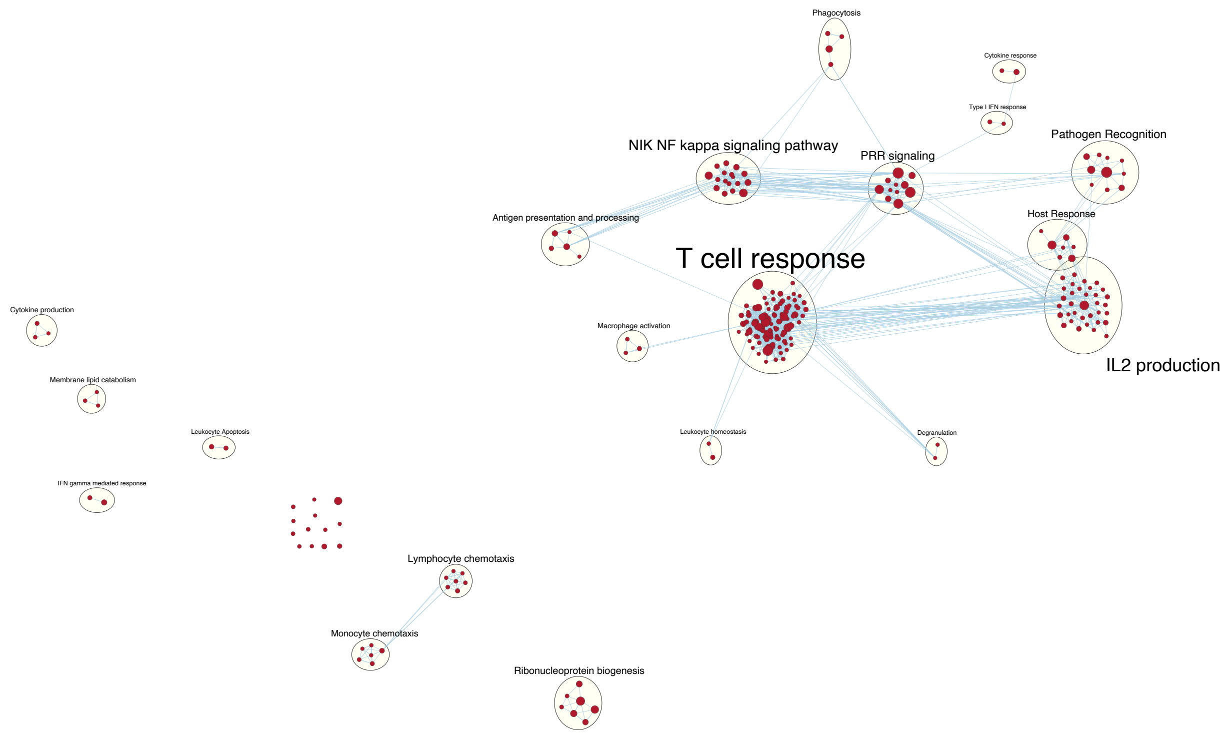

Supplementary Figure 3. GSEA delineated biological pathways upregulated in the bone of *M. avium* infected mice. Cytoscape and Enrichment Map were used for the visualization of the GSEA results as a network of enriched sets (FDR Q <0.05). Nodes representing enriched gene sets are goupr and annotated by their similarity according to the related gene sets. Node size is proportional to the total number of genes within each gene set. Proportion of shared genes between gen sets is represented as the thickness of the red line between nodes.

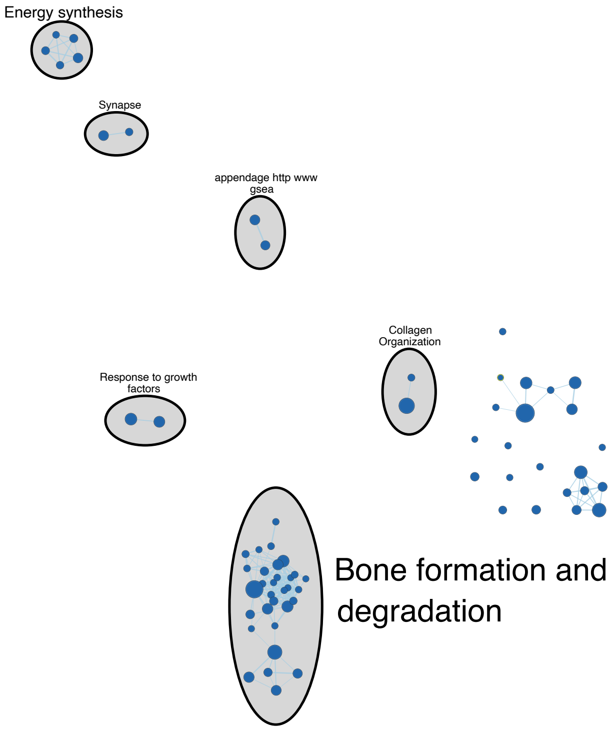

Supplementary Figure 4. GSEA delineated biological pathways downregulated in the bone of *M. avium* infected mice. Cytoscape and Enrichment Map were used for the visualization of the GSEA results as a network of enriched sets (FDR Q <0.05). Nodes representing enriched gene sets are goupr and annotated by their similarity according to the related gene sets. Node size is proportional to the total number of genes within each gene set. Proportion of shared genes between gen sets is represented as the thickness of the red line between nodes.
